# Recent advances in the improvement of genetic resistance against disease in vegetable crops

**DOI:** 10.1093/plphys/kiae302

**Published:** 2024-05-26

**Authors:** William J W Thomas, Junrey C Amas, Aria Dolatabadian, Shuanglong Huang, Fangning Zhang, Jaco D Zandberg, Ting Xiang Neik, David Edwards, Jacqueline Batley

**Affiliations:** School of Biological Sciences, The University of Western Australia, Perth, 6009, Australia; School of Biological Sciences, The University of Western Australia, Perth, 6009, Australia; School of Biological Sciences, The University of Western Australia, Perth, 6009, Australia; Department of Plant Science, University of Manitoba, Winnipeg, R3T 2N2, Canada; College of Life Sciences, Shandong Normal University, Jinan, 250014, China; School of Biological Sciences, The University of Western Australia, Perth, 6009, Australia; Department of Biological Sciences, National University of Singapore, Singapore, 117543, Republic of Singapore; NUS Agritech Centre, National University of Singapore, Singapore, 118258, Republic of Singapore; School of Biological Sciences, The University of Western Australia, Perth, 6009, Australia; Centre for Applied Bioinformatics, The University of Western Australia, Perth, 6009, Australia; School of Biological Sciences, The University of Western Australia, Perth, 6009, Australia

**Advances**

- Many *R* genes have been cloned and functionally validated. This has contributed to our understanding of *R* gene mechanisms while providing effective resistance.
- Pangenomes provide the most accurate representation of a species genome by incorporating genetic diversity from a range of individuals. The number of pangenomes for vegetable crops is growing.
- Developments in resistance gene enrichment sequencing (RenSeq), such as SMRT RenSeq, have facilitated the rapid identification of causal *R* genes, as well as characterizing the species-wide repertoires of *R* genes.
-omics technologies have provided some insights into the complex mechanisms of quantitative resistance (QR).
- Alternative biotechnologies, such as ncRNA, can be used to enhance disease resistance.

## Introduction

Vegetable crops are of immense importance due to their nutritional value (Slavin and Lloyd 2012), environmental sustainability (Parajuli et al. 2019), contribution to food security (Bokelmann et al. 2022), and economic and cultural significance (Tonini et al. 2022). They are found in several plant families and are cultivated for their edible flower buds, fruit, leaves, stems, and tubers. The diversity of vegetable crops is no better illustrated than by *Brassica rapa* and *B. oleracea*, 2 species whose many morphotypes account for more than 30 household vegetables, including broccoli, bok choy, cabbage, and cauliflower, among many others. The major vegetable crops include potato, tomato, cucumber, eggplant, cabbage, lettuce, and broccoli (FAO 2023). The worldwide production of vegetables was 1.15 billion metric tons in 2021, with countries such as China, India, the United States, Turkey, and Iran contributing significant yields (FAO 2023). Among the many challenges facing vegetable growers, diseases caused by microbes pose a serious threat to both crop yield and quality (Richard et al. 2022). Disease-related yield loss in vegetables is considerable, with an estimated loss of more than 20% in major crops like potato (Savary et al. 2019) and reports as high as 40% in other vegetables (Gupta and Thind 2018). The diversity of vegetable-infecting pathogens is extensive, with numerous types of bacteria, fungi, oomycetes, protists, and viruses responsible for disease (Supplementary Table S1). These pathogens have a profound impact on virtually all aspects of plant health, including interfering with physiological processes, hindering the uptake of nutrients, and weakening structures (Baligar et al. 2007; Yang and Luo 2021). As a result, the identification of naturally occurring genetic resistance has been an important objective among researchers.

Plants have developed intricate defense mechanisms to combat these pathogens and maintain their survival. Together with pathogens, they have evolved a complex network of highly specialized interactions leading to a pattern of mutual selection and co-evolution (Zhang 2023). In brief, plants have evolved an innate immune system to respond to pathogens, employing different molecular defense mechanisms. Pathogen-associated molecular pattern (PAMP)-triggered immunity (PTI) and effector-triggered immunity (ETI) are 2 important pathways within the immune system (Jones and Dangl 2006). PTI is initiated when the cell surface's pattern recognition receptors (PRRs) detect PAMPs, or in some cases damage-associated molecular patterns from cell wall degradation (Zhou et al. 2020). The activation of PTI does not typically cause complete host cell death. However, some pathogens can overcome resistance by secreting effector proteins, which facilitate pathogenesis by weakening the host and/or suppressing PTI (Thomma et al. 2011). In response, plants have evolved nucleotide-binding oligomerization domain-like receptors (NLRs) and transmembrane leucine-rich repeat receptors (TM-LRRs) to recognize these effectors. TM-LRRs can generally be further classified into 2 main groups: receptor-like proteins (RLPs) and receptor-like kinases (RLKs). NLRs detect effectors intracellularly, while RLPs and RLKs are cell-surface localized and recognize effectors extracellularly (Bi et al. 2021). Successful effector recognition by these proteins leads to ETI. Additionally, infected plants can generate mobile signals that induce systemic acquired resistance throughout the plant, protecting against future infections (Klessig et al. 2018).

Qualitative resistance is race specific and involves the recognition of specific pathogen effectors by plant proteins encoded by resistance genes (*R* genes). This type of *R* gene-mediated resistance typically manifests as a robust immune response characterized by localized cell death, known as a hypersensitive response (HR). However, not all *R* gene-mediated resistance results in HR (Balint-Kurti 2019). In contrast, quantitative resistance, also referred to as partial or race-nonspecific resistance, is thought to be controlled by many genes of minor effect. ETI corresponds to qualitative resistance, while PTI may contribute toward quantitative resistance. Recently, several notable studies have shed light on the interplay between PTI and ETI during pathogen infection (Ngou et al. 2021; Pruitt et al. 2021; Tian et al. 2021; Yuan et al. 2021a). These studies collectively demonstrated that major components in both PTI and ETI are essential for effective resistance. Yuan et al. (2021a) and Ngou et al. (2021) provided compelling evidence that PRRs play an important role in intracellular defense, as mutants lacking specific PRRs showed an impaired HR induced by bacterial effectors. Pruitt et al. (2021) and Tian et al. (2021) focused on the involvement of key components in the ETI pathway, such as EDS1, PAD4, SAG101, and helper NLRs. While these components are known to be important for ETI, their role in PTI was less understood. Pruitt et al. (2021) demonstrated that mutants lacking EDS1 and PAD4 exhibited reduced ethylene production, reactive oxygen species generation, and callose deposition during PTI activation. These findings highlight the interconnectedness of PTI and ETI and how they reinforce each other to enhance plant defense against invading pathogens.

Climate change has been linked to fostering pathogens and diseases. Rising temperatures, altered precipitation patterns, and changes to other environmental parameters create favorable conditions for the proliferation and spread of disease-causing pathogens (Pokhrel 2021). Climate change also influences the physiology of plants, making them more susceptible to infection (Becklin et al. 2016). The complex interactions between the changing climate, plants, and their pathogens further emphasize the need for proactive measures to mitigate the impact of disease on plant health and ensure sustainable crop production. Developing climate-resilient crop varieties with enhanced disease resistance involves harnessing genetic diversity within crop species and introgressing beneficial traits from their wild relatives (Rajpal et al. 2023).

Significant progress in plant genomics, and more recently pangenomics, has enabled the exploration of vegetable genome diversity and helped decipher it for improved resistance outcomes. These advances have underpinned analyses such as quantitative trait locus (QTL) mapping and genome-wide association studies (GWAS) to pinpoint genomic regions and genes associated with disease resistance (Razzaq et al. 2021). The influx of high-quality sequencing data for vegetable crops sets the stage for the largescale identification of the genomic basis of resistance. This will in turn improve our understanding of the molecular mechanisms that underpin the interaction between plants and their pathogens. Furthermore, manipulating resistance through genetic engineering is now feasible (Dong and Ronald 2019). In this review, we highlight and offer insights into the recent advances in disease resistance studies in vegetables within the past 5 years, covering species that are primarily grown and consumed as vegetables.

## Recently cloned *R* genes

The impact and sheer diversity of diseases that infect vegetable species has warranted significant research into the identification of effective resistance. Since the first vegetable *R* gene was identified in 1993, the NLR *Pto* in tomato (Martin et al. 1993), numerous disease resistance QTL, candidate genes, and functionally validated *R* genes have been identified, mapped, and cloned. Despite the identification of *Cf9*, a TM *R* gene in tomato, which was published the following year (Jones et al. 1994), NLRs have been the main focus of *R* gene identification efforts in vegetable crops, with little research devoted to TM receptors. The breadth of *R* gene discoveries made in vegetables over the past 30 years is too vast to comprehensively cover in this review, so here we have focused on recently cloned *R* genes (Table 1).

**Table 1.** Recently cloned *R* genes in vegetable crops

Family	Species	Vegetable	Pathogen	Pathogen type	Disease	Cloned *R* gene	*R* gene type	Resistance mechanism	Cloning method	References
Brassicaceae	*Brassica juncea*	Mustard greens	*Albugo candida*	Basidiomycete	White rust	*BjuWRR1*	NLR (CNL)	Resistance against 6 *A. candida* isolates	Map-based cloning	Arora et al. (2019b)
Solanaceae	*Solanum lycopersicum*	Tomato	*Fusarium solani*, *F. oxysporum*, *Verticillium dahliae*, *Clavibacter michiganense subsp*. *michiganense*, *and Ralstonia solanacearum*	Fungus or bacteria	Five soil-borne diseases	*SlTLP5, SlTLP6*	TLP	Broad-spectrum resistance via enhancing β-1,3-glucanase activity	Overexpression and CRISPR/Cas9 knockout	Li et al. (2023)
	*Solanum lycopersicum*	Tomato	*Tospoviruses* spp.	Virus	Tomato spotted wilt virus, groundnut ring spot virus, tomato chlorotic spot virus	*Sw-5b*	NLR (CNL)	Broad-spectrum resistance via recognition of PAMP-like region within viral effector	Map-based cloning	Zhu et al. (2017)
	*Solanum lycopersicum*	Tomato	*Tomato yellow leaf curl virus*	Virus	Tomato yellow leaf curl virus	*Ty-2*	NLR (CNL)	Race-specific resistance towards TYLCV-IL and TYLCV-[CN: SH2]) strains	Map-based cloning	Shen et al. (2020)
	*Solanum americanum*	Potato wild relative	*Phytophthora infestans*, *P. parasitica*, *and P. palmivora*	Oomycete	Potato late blight, tobacco black shank and cacao black pod disease	*Rpi-amr3*	NLR (CNL)	Race-specific resistance towards *AVRamr3* and its homologs in other *Phytophthora* spp.	Single-molecule real-time resistance gene enrichment sequencing (SMRT RenSeq)	Witek et al. (2016), Lin et al. (2022)
	*Solanum americanum*	Potato wild relative	*Phytophthora infestans*	Oomycete	Potato late blight	*Rpi-amr1*	NRC helper-dependent NLR (CNL)	Broad-spectrum resistance against 19 *P. infestans* isolates	SMRT RenSeq and association genetics	Witek et al. (2021)
	*Solanum americanum*	Potato wild relative	*Phytophthora infestans*	Oomycete	Potato late blight	*Rpi-amr4, R02860, R04373*	NLR (CNL)	Race-specific resistance towards *PITG_22825* (*AVRamr4*), *PITG_02860* and *PITG_04373*	Pan-NLRome construction, bulked segregant analysis RenSeq and map-based cloning	Lin et al. (2023)
	*Solanum chacoense, S. berthaultii, S. tarijense*	Potato wild relatives	*Phytophthora infestans*	Oomycete	Potato late blight	*Rpi-chc1.1, Rpi-chc1.2*	NLR (CNL)	Race-specific resistance towards *Avrchc1.1* and *Avrchc1.2*	Map-based cloning	Monino-Lopez et al. (2021)
	*Solanum stoloniferum*	Potato wild relative	*Potato virus Y*	Virus	Potato virus Y	Ry*_sto_*	NLR (TNL)	Extreme resistance to potato virus Y	SMRT RenSeq	Grech-Baran et al. (2020)
	*Capsicum annuum*	Capsicum/bell pepper	*Phytophthora infestans*	Oomycete	Potato late blight	Ca*Rpi-blb2*	NLR (CNL)	Race-specific resistance towards Avrblb2 family effectors in non-adapted host	Reverse genetics in *Nicotiana benthamiana*	Oh et al. (2023)

Abbreviations: CNL, coiled-coil nucleotide-binding site leucine-rich repeat; NLR, nucleotide-binding site leucine-rich repeat; NRC, NLR required for cell death; TLP, thaumatin-like protein; TNL, Toll/Interleukin 1 nucleotide-binding site leucine-rich repeat; WAKL, wall associated kinase-like.

Vegetable crops within the Solanaceae family represent the most significant progress in *R* gene identification and cloning, especially potato (Yuen 2021). Since the Irish potato famine, breeding for potato late blight resistance has been a priority, resulting in more than 70 *R* genes being characterized. For a comprehensive overview of late blight *R* genes, the reader is directed to Paluchowska et al. (2022). Resistance gene enrichment sequencing (RenSeq) was first utilized in potato and has become a staple tool for *R* gene discovery in Solanaceous crops, massively expediting the lengthy process of *R* gene identification and cloning (discussed in more detailed below). Due to the clonal nature of potato cultivation and also because the late blight pathogen *Phytophthora infestans* rapidly overcomes resistance, it is no surprise that the majority of recently cloned *R* genes were sourced from potato wild relatives (Paluchowska et al. 2022). The successful identification and usage of *R* genes from these uncultivated species highlights the significant advances in plant genomics and resistance breeding. Oh et al. (2023) expanded this concept further by exploring potato late blight resistance in pepper (*Capsicum annuum*), a nonhost species. Using *Nicotiana benthamiana* as a surrogate host, they identified pepper NLRs that are homologous to known late blight receptors from wild potato relatives, which also recognized *P. infestans* and conferred effective resistance. Their results suggested that nonhost NLRs may in fact be more tolerant to effector-mediated immune suppression by non-adapted pathogens compared with NLRs derived from host species, opening the door for nonhost plants to be explored as valuable sources of resistance (Oh et al. 2023).

Of the cloned Solanaceae *R* genes, NLRs are the most typical, with most being coiled-coil nucleotide-binding site-leucine-rich repeats (CNLs) or Toll/Interleukin-1 nucleotide-binding site-leucine-rich repeats (TNLs) (Table 1) (Paluchowska et al. 2022). Despite our understanding of NLR-mediated resistance, recent research has unveiled new mechanisms of NLR resistance that go against the traditional definitions of plant ETI. In tomato, the CNL *Sw-5b* was demonstrated to confer broad-spectrum resistance against American-type tospoviruses by recognizing a conserved PAMP-like region present within a viral movement protein (Zhu et al. 2017). This is similar to how mammalian NLRs (i.e. NACHT-LRRs) function and opposes the idea that NLR-mediated resistance in plants is solely race specific and occurs via ETI (Jones et al. 2016). Another example is the potato late blight *R* gene *Rpi-amr1*, an NRC-dependent (NLR required for cell death) CNL, which was also found to display broad-spectrum resistance against 19 *P. infestans* isolates (Witek et al. 2021). These novel findings are timely because there is growing evidence that PTI and ETI are not 2 distinct layers of defense, as was once thought, and our understanding of the crosstalk between them is deepening (Ngou et al. 2021; Pruitt et al. 2021; Tian et al. 2021; Yuan et al. 2021a).

Only 1 *R* gene from the Brassicaceae family has been recently cloned despite the large-scale cultivation of *Brassica* vegetables. Specifically, *BjuWRR1* was identified and cloned in mustard greens and confers white rust resistance (Arora et al. 2019b). The gene was identified through a traditional map-based cloning approach, whereas a range of novel methods have been applied to Solanaceous vegetables, perhaps explaining the greater number of cloned *R* genes in Solanaceae. Therefore, the application of alternative techniques, such as RenSeq and CRISPR/Cas9-based approaches, may be an avenue to expedite *R* gene cloning efforts in *Brassica* vegetables.

For the other plant families that comprise major vegetables, such as Asteraceae, Cucurbitaceae, and Fabaceae, there are recent studies that focus on mapping resistance QTL and *R* genes. The subject of such studies included vegetables such as lettuce (Asteraceae) (Christopoulou et al. 2015; Parra et al. 2021a); pumpkin, squash, and zucchini (Cucurbitaceae) (Holdsworth et al. 2016; Shrestha et al. 2021; Wang et al. 2021b; Alavilli et al. 2022; Xu et al. 2022); and common bean and pea (Fabaceae) (Sun et al. 2016; Goncalves-Vidigal et al. 2020; Williamson-Benavides et al. 2021; Wu et al. 2022). However, despite these efforts, very few *R* genes have been cloned within the past 5 years. This is perhaps a reflection of the less significant economic value of these crops within a global context. The inherent scarcity of *R* genes in Cucurbit vegetables due to the family-wide loss of NLR lineages may explain this shortfall in Cucurbitaceae (Lin et al. 2013; Andolfo et al. 2021). Furthermore, in common bean and pea, research into the control of insects, such as aphids, through genetic resistance appears to be equally important (Javed et al. 2022; Rahman et al. 2023). Recent cloning of *R* genes in vegetable species not belonging to Solanaceae is still scarce. Nevertheless, with improved genomic resources and a growing pool of resistance QTL and *R* gene candidates, future cloning will undoubtably increase.

## Genomic resources and *R* gene identification

### Pangenomes

The increasing availability of genomic resources for crops has facilitated a deeper understanding of genomic variation while at the same time exposing the inadequacy of single reference genomes in capturing the species-wide genetic landscape (Zanini et al. 2022). As a result, there has been a surge in the development of pangenomes, which have emerged as a powerful resource for studying widescale genomic architecture because they incorporate multiple individuals (Golicz et al. 2020). Pangenomes have unlocked new perspectives on genetic variation and are now considered to be the most comprehensive reference (Bayer et al. 2020). Within a pangenome, genes can be classified as core (those that are present in all the individuals) or dispensable (those that are present in only some individuals) (Golicz et al. 2020). In some cases, dispensable genes can be further classified as soft core, shell, or cloud genes depending on their prevalence (Tang et al. 2022). In recent years, several pangenomes have been constructed for vegetable crops, uncovering previously unknown genetic variability that offers tremendous potential for crop genomic studies and breeding (Table 2).

**Table 2.** Vegetable pangenomes and genetic variants identified through pangenomic analysis

Vegetable (species)	Construction method	Genetic variants identified	No. of individuals	References
Potato (*Solanum tuberosum*)	Iterative mapping	Predicted genes ranged from 44,859 to 88,871 for each cultivar/accession, of which 13,123 were core genes (present in all 45 accessions); 5,743 were softcore (present in 42 to 44 accessions); 28,471 were shell clusters (found in 2–41 accessions); 4,064 were accession specific	45	Tang et al. (2022)
Pepper (*Capsicum anuum*)	Iterative mapping	Identified 89,181 total genes, of which 28,840 are core genes	383	Ou et al. (2018)
Tomato (*Solanum lycopersicum*)	Iterative mapping	Identified 238,490 SVs with different accessions containing between 1,928 and 45,840 SVs	100	Alonge et al. (2020)
	Graph assembly	Identified 51,155 genes, of which 14,507 are dispensable genes	131	Zhou et al. (2022)
Eggplant (*Solanum melongena*)	Iterative mapping	Identified 35,732 genes, of which 31,424 are core genes, 922 are softcore genes (shared by 25 accessions), 1,556 are shell genes (shared by 2 to 24 accessions), and 1,246 are accession specific	26	Barchi et al. (2021)
Cucumber (*Cucumis sativum*)	Graph pangenome	Identified 22,822 genes, of which, 18,651 are core genes and 8,171 are dispensable genes. A further 54,107 SVs were also detected	12	Li et al. (2022)
Pea (*Pisum sativum*)	Iterative mapping	Identified 15,470 core genes, 6,170 soft-core genes, 41,028 shell genes, and 50,108 cloud genes	118	Yang et al. (2022)
Cabbage, broccoli, cauliflower and kohlrabi (*Brassica oleracea*)	Iterative mapping	Identified 58,315 genes, of which 45,961 are core genes and 12,354 are dispensable genes	87	Bayer et al. (2021)
Turnip, bok choy, pak choi and Chinese cabbage (*Brassica rapa*)	Iterative mapping	Identified 59,864 genes, of which 39,952 are core genes and 19,912 are dispensable genes	77	Bayer et al. (2021)
Ethiopian mustard (*Brassica carinata*)	Iterative mapping	Identified 127,421 genes, of which 88,307 are core genes, 21,262 softcore, 16,852 shell, and 65,792 cloud genes	82	Niu et al. (2024)
Asparagus bean (*Vigna unguiculata* ssp. *sesquipedialis*)	De novo assembly	Identified 20,336 core genes, 6,507 dispensable genes, and 2,004 accession-specific genes	4	Liang et al. (2022)

Pangenomes facilitate the detection of structural variations (SVs) such as presence-absence variations (PAVs) and copy number variations, which have been increasingly recognized as impacting phenotypes (Yuan et al. 2021b). For example, pangenome studies have uncovered extensive SVs affecting disease resistance traits in *Brassica* vegetables (Bayer et al. 2021). In the *B. rapa* pangenome, 30% of *R* genes were affected by PAV (Amas et al. 2023). Several of these genes co-localized with genomic regions controlling resistance to important diseases, including clubroot, downy mildew, and turnip mosaic virus, indicating their possible involvement in conferring resistance. Similarly, in the pangenome of *B. oleracea*, dispensable genomic regions were found to be enriched with *R* genes, some of which were associated with stem rot and blackleg resistance (Golicz et al. 2016; Bayer et al. 2019). These studies suggest that the dispensable genome contains valuable *R* genes that are absent from single references, emphasizing the importance of pangenomes for candidate gene identification.

In vegetable crops, the utilization of pangenomes is a relatively recent advancement, and there is limited progress in its utility for isolating *R* genes. However, the application of pangenome analysis has proven beneficial in other crops such as wheat. Notably, the wheat pangenome was essential in the identification and isolation of *YR63*, a broad-spectrum *R* gene effective against multiple races of the wheat stripe rust pathogen (Mackenzie et al. 2023). The gene was previously unresolved in 2 single genome references but was correctly mapped in the pangenome, which was essential for cloning. While no *R* genes have been directly isolated from vegetable pangenomes yet, the above example highlights the potential value pangenomic analysis adds toward the identification of causal *R* genes. Furthermore, pangenomes have also been used to uncover other genetic factors involved in disease progression, such as loss-of-susceptibility *R* genes. For example, the cucumber pangenome constructed from 12 varieties enabled the identification of 3 susceptibility transcription factors that enhance gray mold disease (Liu et al. 2024). This highlights the wider applicability of pangenomes in characterizing genetic factors beyond traditional *R* genes. In addition, pangenomes have the potential to complement traditional gene mapping approaches, such as linkage analysis, by capturing the broader genomic information of a species (Amas et al. 2023). The increasing availability of pangenomes for vegetables means they can be leveraged to bolster the identification of agronomically important *R* genes and genetic factors controlling disease resistance in these crops.

As the field of pangenomics continues to evolve, new construction methods have emerged. The 3 most widely utilized methods include iterative, de novo, and graph assembly (Fernandez et al. 2022). Graph pangenomes are a recent development, integrating the features of both iterative and de novo assembly (Eizenga et al. 2020) (Box 1). Currently, graph pangenomes have been constructed for 2 key vegetable crops: tomato and cucumber (Li et al. 2022; Zhou et al. 2022). The tomato graph pangenome was used to uncover missing heritability of economically important traits, providing a promising approach for understanding the role of genome structure in heritable traits (Edwards and Batley 2022; Zhou et al. 2022). Moreover, the cucumber pangenome facilitated the identification of SVs affecting fruit quality and yield associated traits, enabling the implementation of a highly accurate and cost-effective SV-based genotyping strategy in cucumber breeding (Li et al. 2022). The development of pangenomes, and in particular graph pangenomes, may be fundamental in unravelling the complex genomic basis of important traits. The application of pangenomes to investigate the genetic variation responsible for disease resistance has the potential to revolutionize crop breeding programs.

**Box 1. Graph pangenomes**

Advances in pangenome development, including graph pangenome construction, are revolutionizing genomic-based studies by enabling a comprehensive and dynamic representation of genetic diversity, thereby enhancing our understanding of complex traits, including disease resistance. Graph pangenomes offer several advantages over traditional pangenomes. For instance, graph pangenomes can better represent complex genetic variations, including large-scale SVs and highly complex genomic regions, than the linear representations used in traditional pangenomes (Zhou et al. 2022). They can also facilitate a seamless integration of new data, including epigenomic and transcriptomic datasets, making room for continuous improvements as more genomes become available (Zanini et al. 2022).

Graph pangenomes are also more efficient than traditional pangenomes in terms of storing data. The graph approach can compress redundant copies of analogous sequences into a smaller data structure but still represent the whole data (Eizenga et al. 2020). This results in reduced storage requirements and enhanced scalability, particularly for species with massive genome sizes, including polyploids. In addition, graph pangenomes can provide a comprehensive framework for improving reference-guided assembly of genomes (Hickey et al. 2024). This enables more accurate read mapping and better reconstruction of genomes, especially with those that have high SVs or repetitive sequences.

Because of these advantages, graph pangenomes are an excellent resource for various applications. For example, graph pangenomes are highly beneficial for population studies. By integrating diverse genomes from a population into a unified representation, they can capture the full spectrum of genomic variation within the population, enabling comprehensive comparative analyses among individuals within that population (Hübner 2022). Ancestral genomes can also be efficiently reconstructed using the graph pangenome framework, which then allows for deciphering evolutionary events, including gene loss, gain, and transfer, providing better evolutionary insights on the population (Muffato et al. 2023).

Lastly, graph pangenomes show immense potential for accelerating crop breeding programs. As mentioned above, they allow for the identification of more genetic variants including those highly affected by SVs and facilitate discovery of rare and novel variations (Zhou et al. 2022). This enhances the development of molecular markers that can be used for trait mapping, MAS, and GS (Usadel 2022). Furthermore, using the comprehensive information from the graph pangenome, plant breeders can make better decisions on selecting parental lines, designing crosses, and predicting the performance of the progenies. These ultimately assist in expediting the process of developing superior cultivars with desirable traits including disease resistance.

### Resistance gene enrichment sequencing

RenSeq is a targeted sequencing approach that reduces genome complexity by enriching and sequencing NLR genes within a genome (Jupe et al. 2013). Its development in potato marked a significant milestone in accessing the genome-wide repertoire of NLRs without the cost of whole-genome sequencing. Since its inception, RenSeq has been combined with various technologies and analyses to facilitate the rapid cloning of *R* genes in plants (Zhang et al. 2020; Bohra et al. 2022). The combination of RenSeq and association genetics gave rise to AgRenSeq, which enabled the exploration and cloning of NLRs in wheat without the need for a reference genome (Arora et al. 2019a). Loss-of-function mutagenesis together with RenSeq, termed MutRenSeq, facilitated the cloning of 2 wheat stem rust *R* genes (Steuernagel et al. 2016). Another spin-off, SMRT RenSeq, which couples RenSeq with long-read sequencing using single-molecular real-time (SMRT) sequencing by PacBio, holds great promise (Witek et al. 2016). SMRT RenSeq has the advantage of sequencing *R* genes in their entirety, yielding accurate full-length sequences and thereby avoiding issues with fragmented assembly that so often complicate NGS-based RenSeq (Witek et al. 2016). SMRT RenSeq in *Solanum americanum*, a potato wild relative, led to the cloning of 4 *R* genes that recognize late blight, namely *Rpi-amr1* (Witek et al. 2021), *Rpi-amr4*, *R02860*, and *R04373* (Lin et al. 2023). Furthermore, SMRT RenSeq was pivotal in cloning the potato virus Y resistance gene *Ry_sto_* in *S. stoloniferum* (Grech-Baran et al. 2020). Despite these successes, the bioinformatics expertise needed to carry out the RenSeq workflow stands as a barrier to its more widespread uptake. Adams et al. (2023) recently developed HISS, which is a python-based unified workflow for both SMRT RenSeq and AgRenSeq that attempts to reduce the steep learning curve of RenSeq analysis. As the accessibility of long-read sequencing increases, advances such as HISS will be imperative in propelling RenSeq and SMRT RenSeq as staple tools for novel *R* gene discovery in vegetable crops.

In addition to pinpointing and cloning specific *R* genes, RenSeq has enabled the genome-wide characterization and evolutionary analysis of NLR genes for various species, thereby defining their NLRome. This was first carried out in tomato, which resulted in 105 novel NLRs being identified (Andolfo et al. 2014). More recent studies which use SMRT RenSeq, have categorized genome-wide NLR diversity in the wild grass *Haynaldia villosa* (Huang et al. 2022) and in winter wheat (Kale et al. 2022). Although there are bioinformatic pipelines that predict NLR loci within a reference genome, for example, NLRannotator (Steuernagel et al. 2020) and NLGenomeSweeper (Toda et al. 2020), the RenSeq-based approach enables exploration of species that have either low-quality or incomplete references or that lack one entirely (Tirnaz et al. 2022). Furthermore, in silico prediction is limited to those varieties that have reference genomes, while RenSeq can be performed in a diverse range of individuals to more cost-effectively capture species-wide NLR diversity. This benefit was exemplified in *Arabidopsis thaliana* where SMRT RenSeq was used to define the pan-NLRome by isolating the NLR complement of 64 different accessions (Van de Weyer et al. 2019). By incorporating multiple carefully chosen accessions with different geographic origins, the authors were able to measure NLR diversity on an unprecedented scale, determine how this diversity related to important pathogens, and characterize patterns of SV (Van de Weyer et al. 2019). Recently, a pan-NLRome was generated for *S. americanum* that enabled the rapid cloning of 3 late blight *R* genes and uncovered a more detailed understanding of the resistance landscape between *S. americanum* and *P. infestans* (Lin et al. 2023). Pan-NLRomes, and possibly in the future pan-RGAomes (Box 2), which are based on the principles of pangenomics, represent the most comprehensive atlas of a species' *R* genes to date. SMRT RenSeq is currently the tool of choice for developing pan-NLRomes, and as it becomes more accessible, so too does the potential for generating pan-RGAomes for a range of vegetables, which will vastly expand our understanding of the *R* genes that protect vegetable crops.

**Box 2. Pan-RGAomes**

Pan-NLRomes have been generated using RenSeq; however, these only capture NLR type *R* genes. Although NLRs are commonly involved in recognizing pathogen effectors and triggering ETI, they are not the only type of *R* gene responsible for resistance. For example, TM-LRRs, which largely comprise RLPs and RLKs, are involved in PTI or *R* gene-mediated resistance against apoplastic fungal pathogens. They have been identified as functional *R* genes in a number of plant-pathogen interactions (Borhan et al. 2022). Lin et al. (2020) carried out RLP/KSeq, a modified version of RenSeq which instead targets RLP/Ks, demonstrating that the methodology underpinning RenSeq can be tweaked to capture different target sequences. By combining the 2 approaches, the genome-wide complement of resistance gene analogues (RGAs), which includes NLRs, RLPs, and RLKs, can be defined. As an expansion of the pan-NLRome, which covers additional types of *R* genes, we proposed this relatively new concept be called the pan-RGAome (Zhang et al. 2020). If new types of *R* gene families are discovered to be involved in pathogen recognition, then we postulate that RenSeq can be modified accordingly to capture those new genes, thereby providing limitless potential for the pan-RGAome to reflect the full complement of a species *R* genes.

## Quantitative resistance

In parallel to the research into qualitative resistance, which has resulted in the cloning of many *R* genes, quantitative resistance (QR) has long been investigated as a complementary source of resistance (Poland et al. 2009). QR, where phenotypes display continuous variation between susceptibility and resistance, is thought to be governed by many genes of small effect (St. Clair 2010; Niks et al. 2015). Compared with qualitative resistance, QR is generally considered more durable, although not completely protected from resistance breakdown (Cowger and Brown 2019). The mechanisms driving polygenic QR are still largely unknown because of the difficulty in teasing apart the network of causal genes underpinning resistance (Corwin and Kliebenstein 2017). Despite this, there have been extensive efforts to map QR QTL in vegetable crops that can be utilized in resistance breeding through marker-assisted selection (MAS) and genomic selection (GS). For example, 4 recent studies in lettuce identified QTL for QR against downy mildew, bacterial leaf spot, grey mold, lettuce drop, and Verticillium wilt (Parra et al. 2021b; Pink et al. 2022; Simko et al. 2022; Sthapit Kandel et al. 2022). The QTLs identified in these studies explained between 6.7% and 36% of the phenotypic variation observed. As reflected by these percentages, one of the major challenges in QR research is accounting for the unidentifiable portion of phenotypic variance that is presumably controlled by other genes that have minor and undetectable effects (Corwin and Kliebenstein 2017). Two variables largely contributing toward this are precision in phenotyping and population size. Depending on the pathogen, phenotyping for QR is often troublesome because it differs with environment, can be easily masked by *R* genes, and in some cases can only be assessed in the field (Yates et al. 2019). The size of QR mapping populations is also an important consideration that influences mapping power. Multiparent advanced generation intercross (MAGIC) populations have been proposed as a way of alleviating issues with population size (Corwin and Kliebenstein 2017). However, only a handful of MAGIC populations have been developed for vegetables, including 2 tomato (Pascual et al. 2015; Campanelli et al. 2019) and 1 more recent eggplant population (Mangino et al. 2022). Rojas et al. (2019) undertook a different approach to further dissect QR by investigating disease symptoms in different plant organs in potato. Using GWAS, they identified 6 leaf-specific and 10 stem-specific QTL conferring QR to late blight, which explained high levels of phenotypic variance (13.7% to 50.9%). Therefore, examining QR under an organ-specific lens may yield a more comprehensive overview of how plants respond to pathogens and how this response varies in different areas of the plant. QTL identification for QR in vegetables is in full motion but comes with inherent challenges that hinder rapid progress, such as unidentifiable genes that are probably contributing toward resistance and the optimization of mapping parameters. Each new study that identifies QR QTL adds 1 more piece to solving the complicated QR puzzle.

Many vegetable QR studies identify genes underlying QTL regions. These can include canonical *R* genes but more often than not include non-*R* genes, which impact plant defense by means other than pathogen recognition (Niks et al. 2015). One notable example of a definite QR gene is the *STAYGREEN* gene in cucumber, which provides QR against 3 different pathogens via a loss-of-susceptibility mechanism (Wang et al. 2019). The authors identified a nonsynonymous mutation in *STAYGREEN* in a resistant cucumber line (Gy14) that resulted in QR against downy mildew, bacterial angular leaf spot, and fungal anthracnose. The prevention of reactive oxygen species overaccumulation in Gy14 was proposed as the most likely mechanism explaining *STAYGREEN*-mediated QR (Wang et al. 2019). As more candidate genes for QR are identified in vegetables, research is extending beyond genomics by incorporating -omics-based technologies to understand their potential involvement in QR (Shaw et al. 2022). Transcriptomics, proteomics, and metabolomics are 3 areas of study being applied to scrutinize the dynamic role of defense genes during pathogen infection, which contributes to our understanding of QR (Wang et al. 2021a). Transcriptomic and proteomic analysis of potato roots infected with the protist pathogen powdery scab revealed that resistant plants had upregulated glutathione metabolism, which is involved in plant redox and immune signaling (Balotf et al. 2022). Lan et al. (2019) employed isobaric tags for relative and absolute quantitation–based proteomic analysis to identify differentially abundant proteins in the roots of Chinese cabbage when challenged with clubroot. The proteins of interest were involved in cytokinin signaling or arginine biosynthesis pathways, both of which are responses to pathogen invasion (Lan et al. 2019). Another study in Chinese cabbage incorporated metabolome profiling in addition to RNAseq to identify 6 pathways involved in clubroot resistance (Wei et al. 2021). In pea, targeted proteomics combined with data-independent acquisition analysis was utilized to propose a defense mechanism against *Ascochyta* blight (Castillejo et al. 2020). These studies exemplify an increasing use of -omics technologies to explore disease resistance mechanisms in vegetable crops. Another study delved into QR against bacterial wilt in tomato, using metaRNAseq to examine resistance within the context of growth-defense trade-offs (Meline et al. 2023). The authors found that when infected, resistant plants executed defense-related pathways while simultaneously allocating resources toward root growth and suppressing genes that negatively regulate water stress tolerance. Their findings suggest that growth-defense trade-offs can be more complex than the assumed antagonism (Meline et al. 2023). Shedding light on the molecular interaction between plants and pathogens enhances our understanding of resistance pathways and the underlying genes that could be potential candidates for QR. It is clear that a holistic approach is necessary to fully appreciate the complexities of QR. Furthermore, the broadening range of -omics technologies used to examine vegetable-pathogen interactions, for example sRNAomics, RNA degradomics, and hormonomics (Stare et al. 2019), could provide novel tools to fill gaps in QR knowledge.

## Alternative biotechnologies

In addition to the advances discussed above, there have been several novel biotechnologies applied to enhance resistance against pathogens, offering alternative avenues for disease resistance improvement in vegetable crops. Inside the eukaryotic genome, noncoding RNAs (ncRNAs), comprising different types based on their length and mode of biogenesis, have been found to play crucial roles in plant immunity (Song et al. 2021). RNA interference (RNAi) mediated by small interfering RNA (siRNA) is one of the major defense mechanisms against pathogens in plants. siRNAs, typically double-stranded RNAs 20 to 24 nucleotides (nt) in length, are derived from either the plant genome or exogenous pathogen RNA sequences or transcripts (Bernstein et al. 2001). The bi-directional movement of siRNAs, functioning as exogenous regulators, between host plants and pathogens has been identified as involved in regulating the expression of target genes (Weiberg et al. 2013; Zeng et al. 2019). Based on this cross-kingdom RNAi mechanism, the application of topical sprays of dsRNA has been explored as a method to control disease by gene silencing, known as spray-induced gene silencing (SIGS). For example, SIGS has been applied in pepper, tomato, and mustard greens to provide resistance against Phytophthora blight, tomato wilt, and Sclerotinia stem rot, respectively (Cheng et al. 2022; Ouyang et al. 2023; Pant and Kaur 2023). However, RNA uptake efficiency differs between pathogens, so spraying conditions need to be optimized, especially to suppress infection by low-uptake-efficiency pathogens (Qiao et al. 2021). When applied directly on wounded leaf surfaces infected with a fungal pathogen, dsRNA showed stronger and more durable improvements to resistance compared with when sprayed on the pathogen or healthy leaf directly. This may be caused by an efficient absorption via plant cells and secondary amplification machinery (Song et al. 2018). Low-pressure spray and RNA load using carbon nanoparticles or layer doubled hydroxide clay nanosheets may help to enhance RNA delivery efficiency and adhesion (Mitter et al. 2017; Schwartz et al. 2020). Other ncRNAs have been identified as having a role in plant immunity, as detailed in Box 3.

**Box 3. Examples of noncoding RNA in disease resistance improvement**

1. MicroRNAs (miRNAs), usually between 21 and 24 nt long, can trigger the production of phased small interference RNAs (phasiRNAs) from their NLR targets (Fei et al. 2013). Among the many miRNA that are involved in plant immunity (Cao et al. 2016; Song et al. 2021), some are also associated with growth and maintaining yield and are therefore of great interest. For example, the *Brassica* miR1885 was found to dynamically regulate both resistance against TuMV and floral development (Cui et al. 2020). In brief, miR1885 directly targets and silences the TNL domain of the *R* gene *BraTNL*. Specifically, miR1885 peaks at the initiation of floral transition, targets and cleaves the TIR domain-containing *BraTIR1* gene, with *BraTIR1* behaving as a *trans-acting silencing* (*TAS*) gene, triggering the production of tasiRNA. This results in the secondary silencing of *BraCP24* and accelerated flowering. Once infected by TuMV, *BraTNL1* expression was induced independently of miR1885, overcoming miR1885-mediated *R* gene turnover and ultimately accelerating floral transition and mitigating the growth-defense trade-off (Cui et al. 2020). Furthermore, miRNAs have been found in other crops, such as maize and rice, to play key roles in the growth-defense trade-off (Kumar et al. 2022; Qu et al. 2023).
2. Long ncRNAs (lncRNAs) have also been demonstrated to be involved in plant-microbe interactions (Joshi et al. 2016). For example, markers have been developed for *B. napus* which detect resistance against clubroot based on differently expressed lncRNAs (Summanwar et al. 2021). The modification of lncRNAs could also be effective in resistance regulation, for example, the silencing of the antisense transcript of *BrMAPK15*, *MSTRG.19915*, increased downy mildew resistance in *B. rapa* ssp. *pekinensis* (Zhang et al. 2021b). Furthermore, the investigation of late blight resistance in tomato revealed that the interaction between lncRNA and miRNA can regulate expression of pathogenesis-related (PR) and NLR genes, with lncRNA being an endogenous target mimic for miRNAs (Jiang et al. 2019; Hou et al. 2020).
3. Circular RNAs (circRNAs) are covalently closed ncRNAs derived from reverse variable splicing of mRNA precursors. They function as competitive endogenous RNAs (ceRNAs) binding to target miRNA and regulating expression of downstream genes (Song et al. 2021). circRNAs responsive to pathogen infection have been identified in vegetable plants, such as tomato and Chinese cabbage (Chidambara et al. 2022; Liu et al. 2022).

On the post-transcriptional modification level, N6-methyladenosine (m^6^A) is the most prevalent internal mRNA modification across eukaryotic species. Although a negative correlation was found between m^6^A methylation ratio and the number of *R* genes and genome size in an evolutionary context (Miao et al. 2022), m^6^A deposition was positively correlated to plant mRNA abundance (Yue et al. 2022). For some plant RNA viruses, infection is manipulated by mRNA modification, which suggests that m^6^A methylation could play a role in plant–virus interactions. In tomato plants infected with *Pepino mosaic virus* (PepMV), overexpression of the m^6^A writer, slHAKAI, negatively regulated PepMV infection via an autophagy pathway (He et al. 2023). A similar increase in m^6^A methylation was found in virus infected rice (Zhang et al. 2021a). However, in the case of watermelon infected by *cucumber green mottle mosaic virus* (CGMMV), decreased m^6^A methylation levels were detected in resistant watermelon plants (He et al. 2021). These studies indicate an ambiguous mechanism for how m^6^A modification participates in plant–virus interactions and highlight how complicated the antiviral modes can be.

Also on the post-translational level, small peptides (up to 20 amino acids [aa] in length), typically without cysteine, are involved in coordinating the immune response, especially in signaling processes (Hu et al. 2018). Tomato plants treated using CAPE1, a conserved peptide elicitor derived from tomato PR-1, displayed stronger resistance against bacterial speck (Chen et al. 2014). Another kind of small cysteine-rich protein, defensin, consists of 45 to 54 aa. The exogenous spray of defensin-like protein fabatin has been demonstrated to enhance cucumber resistance against *Fusarium oxysporum* (Naguib et al. 2021). Similarly, the suppression of grey mold pathogen *B*. *cinerea* infection was observed on tomato after being sprayed with defensin-derived antifungal peptides (Tetorya et al. 2023).

Plant genome editing is another advanced strategy to improve biotic resistance (Dong and Ronald 2019). CRISPR/Cas (clustered regularly interspersed palindromic repeats/CRISPR-associated protein) systems are one of the most efficient site-specific genome editing approaches (Gao 2021). Among the many different systems developed, CRISPR/Cas9 is the most comprehensively studied. Despite this, there are only a few cases of CRISPR/Cas9 being used to improve vegetable resistance against pathogens, all of which are viral diseases. Potyvirus-resistant plants have been developed in cucumber, tomato, and Chinese cabbage (Chandrasekaran et al. 2016; Yoon et al. 2020; Lee et al. 2023). The gene family of eukaryotic translation initiation factor 4E, *eIF4E*, is tightly associated with recessive *R* genes. Using Chinese cabbage as an example, the *Brassica eIF(iso)4E* gene is associated with 2 broad-spectrum recessive *R* genes, and 3 copies of *eIF(iso)4E* were selected as target sequences with 3 single guide RNAs (sgRNAs) respectively. *eIF(iso)4E* mutant plants displaying resistance against Turnip Mosaic Virus (TuMV) were achieved using CRISPR/Cas9 and indels detected around sgRNA target sites, and a strong negative correlation was identified between TuMV resistance and genome editing frequency (Lee et al. 2023). The target gene *eIF4E* can be modified as a significant host factor in future studies, and the efficiency of resistant breeding could be moderated through regulating target gene editing frequency.

The aforementioned biotechnologies are examples of promising alternative strategies that could be utilized to improve the current prevention and control methods against a wide range of vegetable diseases. The development of RNAi-based and peptide-based fungicides, which can be easily synthesized and applied through spraying, as well as being environmentally friendly, represents a new avenue for the integrated management of vegetable diseases.

## Conclusions

**Figure 1.**
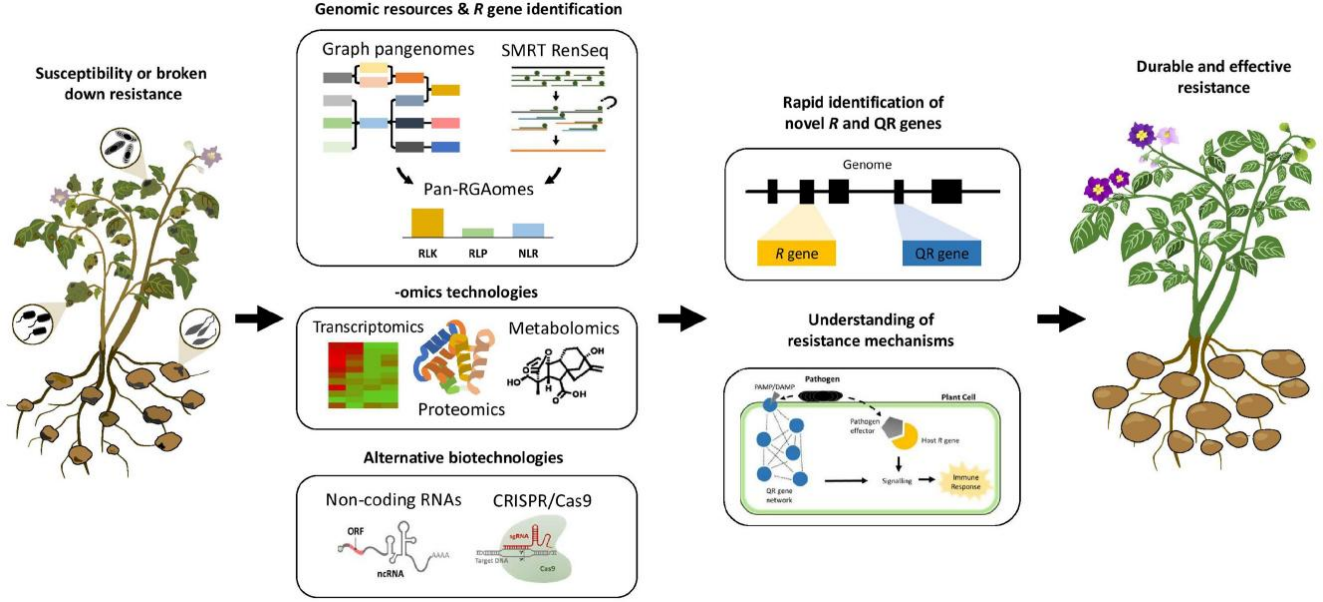
Schematics of the recent advances in the improvement of genetic resistance against disease in vegetables crops. Advances in both pangenomics and RenSeq, specifically the development of graph pangenomes and SMRT RenSeq, will contribute toward generating accurate Pan-RGAomes, which represent the species wide diversity of *R* genes (comprising NLR receptors, RLKs, and RLPs). Different -omics technologies can help to unravel the complex mechanisms driving QR, while the exploitation of CRISPR/Cas9 and noncoding RNAs provides additional routes to enhance disease resistance. Collectively these advances contribute to a deeper understanding of the mechanisms underpinning resistance and enable the identification of novel *R* and QR loci and genes, which translates to durable and effective disease resistance in vegetable crops.

The study of genetic resistance against disease in vegetable crops has seen tremendous progress over the past 3 decades. Research of qualitative resistance has had a head start compared with the more complex QR, which still has many unanswered questions. Harnessing both types of resistance will be key in preventing future resistance breakdown by fast-adapting pathogens. Recent advances in pangenomics, such as graph assembly, and in RenSeq, such as SMRT RenSeq, mean that we are well positioned to exploit the full arsenal of *R* gene diversity. The broadening range of -omics technologies promises to incrementally improve our understanding of the mechanisms behind the plant defense response and QR. Furthermore, alternative biotechnologies will add to the growing toolbox of strategies to manage disease. The recent advances discussed in this review are summarized in Fig. 1. Finally, we put forward some of the challenges facing disease resistance research in vegetable crops (see Outstanding questions). The answers to these questions will fill critical knowledge gaps and will translate into durable and effective disease resistance.

**Outstanding questions**

- How can we best capitalize on the growing pool of resistance QTL and candidate *R* genes to rapidly clone novel *R* genes across all vegetable crops?
- Should disease resistance be a key element of consideration when choosing individuals to incorporate into a pangenome?
- How can the RenSeq pipeline be further simplified? Do any modifications need to be implemented to facilitate the application of RenSeq in vegetables outside of the Solanaceae family?
- How can we exploit the broadening field of multi-omics to gain a more complete understanding of the complex mechanisms underpinning QR?
- How can we optimize RNAi- and peptide-based fungicides so they are effective against all pathogens?

## Supplementary Material

kiae302_Supplementary_Data

## Data Availability

No new data were generated or analyzed in support of this research.

## Supplementary Data

**Supplementary Table S1.** Overview of the common pathogens that cause disease to vegetable crops.

## Dive Curated Terms

The following phenotypic, genotypic, and functional terms are of significance to the work described in this paper:

SAG101 Gramene: AT5G14930
SAG101 Araport: AT5G14930

eIF4E Gramene: AT4G18040
eIF4E Araport: AT4G18040

Cf9 Gramene: AT1G59510
Cf9 Araport: AT1G59510

EDS1 Gramene: AT3G48090
EDS1 Araport: AT3G48090

PAD4 Gramene: AT3G52430
PAD4 Araport: AT3G52430

ETI Gramene: Effector-triggered immunity
ETI Araport: Effector-triggered immunity

PTI Gramene: pattern-triggered immunity
PTI Araport: pattern-triggered immunity

## References

[kiae302-B1] Adams TM , Smith M, Wang Y, Brown LH, Bayer MM, Hein I. HISS: snakemake-based workflows for performing SMRT-RenSeq assembly, AgRenSeq and dRenSeq for the discovery of novel plant disease resistance genes. BMC Bioinformatics. 2023:24(1):204. 10.1186/s12859-023-05335-8 37198529 PMC10193785

[kiae302-B2] Alavilli H , Lee JJ, You CR, Poli Y, Kim HJ, Jain A, Song K. GWAS reveals a novel candidate gene *CmoAP2/ERF* in pumpkin (*Cucurbita moschata*) involved in resistance to powdery mildew. Int J Mol Sci. 2022:23(12):6524. 10.3390/ijms23126524 35742978 PMC9223685

[kiae302-B3] Alonge M , Wang X, Benoit M, Soyk S, Pereira L, Zhang L, Suresh H, Ramakrishnan S, Maumus F, Ciren D, et al Major impacts of widespread structural variation on gene expression and crop improvement in tomato. Cell. 2020:182(1):145–161.e23. 10.1016/j.cell.2020.05.021 32553272 PMC7354227

[kiae302-B4] Amas JC , Bayer PE, Hong Tan W, Tirnaz S, Thomas WJW, Edwards D, Batley J. Comparative pangenome analyses provide insights into the evolution of *Brassica rapa* resistance gene analogues (RGAs). Plant Biotechnol J. 2023:21(10):2100–2112. 10.1111/pbi.14116 37431308 PMC10502758

[kiae302-B5] Andolfo G , Jupe F, Witek K, Etherington GJ, Ercolano MR, Jones JDG. Defining the full tomato NB-LRR resistance gene repertoire using genomic and cDNA RenSeq. BMC Plant Biol. 2014:14(1):120. 10.1186/1471-2229-14-120 24885638 PMC4036795

[kiae302-B6] Andolfo G , Sánchez CS, Cañizares J, Pico MB, Ercolano MR. Large-scale gene gains and losses molded the NLR defense arsenal during the *Cucurbita* evolution. Planta. 2021:254(4):82. 10.1007/s00425-021-03717-x 34559316 PMC8463517

[kiae302-B7] Arora H , Padmaja KL, Paritosh K, Mukhi N, Tewari AK, Mukhopadhyay A, Gupta V, Pradhan AK, Pental D. *BjuWRR1*, a CC-NB-LRR gene identified in *Brassica juncea*, confers resistance to white rust caused by *Albugo candida*. Theor Appl Genet. 2019b:132(8):2223–2236. 10.1007/s00122-019-03350-z 31049632

[kiae302-B8] Arora S , Steuernagel B, Gaurav K, Chandramohan S, Long Y, Matny O, Johnson R, Enk J, Periyannan S, Singh N, et al Resistance gene cloning from a wild crop relative by sequence capture and association genetics. Nat Biotechnol. 2019a:37(2):139–143. 10.1038/s41587-018-0007-9 30718880

[kiae302-B9] Baligar VC , Fageria NK, He ZL. Nutrient use efficiency in plants. Commun Soil Sci Plant Anal. 2007:32(7-8):921–950. 10.1081/CSS-100104098

[kiae302-B10] Balint-Kurti P . The plant hypersensitive response: concepts, control and consequences. Mol Plant Pathol. 2019:20(8):1163–1178. 10.1111/mpp.12821 31305008 PMC6640183

[kiae302-B11] Balotf S , Wilson R, Nichols DS, Tegg RS, Wilson CR. Multi-omics reveals mechanisms of resistance to potato root infection by *Spongospora subterranea*. Sci Rep. 2022:12(1):10804. 10.1038/s41598-022-14606-y 35752627 PMC9233701

[kiae302-B12] Barchi L , Rabanus-Wallace MT, Prohens J, Toppino L, Padmarasu S, Portis E, Rotino GL, Stein N, Lanteri S, Giuliano G. Improved genome assembly and pan-genome provide key insights into eggplant domestication and breeding. Plant J. 2021:107(2):579–596. 10.1111/tpj.15313 33964091 PMC8453987

[kiae302-B13] Bayer PE , Golicz AA, Scheben A, Batley J, Edwards D. Plant pan-genomes are the new reference. Nat Plants. 2020:6(8):914–920. 10.1038/s41477-020-0733-0 32690893

[kiae302-B14] Bayer PE , Golicz AA, Tirnaz S, Chan CK, Edwards D, Batley J. Variation in abundance of predicted resistance genes in the *Brassica oleracea* pangenome. Plant Biotechnol J. 2019:17(4):789–800. 10.1111/pbi.13015 30230187 PMC6419861

[kiae302-B15] Bayer PE , Scheben A, Golicz AA, Yuan Y, Faure S, Lee HT, Chawla HS, Anderson R, Bancroft I, Raman H, et al Modelling of gene loss propensity in the pangenomes of three *Brassica* species suggests different mechanisms between polyploids and diploids. Plant Biotechnol J. 2021:19(12):2488–2500. 10.1111/pbi.13674 34310022 PMC8633514

[kiae302-B16] Becklin KM , Anderson JT, Gerhart LM, Wadgymar SM, Wessinger CA, Ward JK. Examining plant physiological responses to climate change through an evolutionary lens. Plant Physiol. 2016:172(2):635–649. 10.1104/pp.16.00793 27591186 PMC5047093

[kiae302-B17] Bernstein E , Caudy AA, Hammond SM, Hannon GJ. Role for a bidentate ribonuclease in the initiation step of RNA interference. Nature. 2001:409(6818):363–366. 10.1038/35053110 11201747

[kiae302-B18] Bi G , Su M, Li N, Liang Y, Dang S, Xu J, Hu M, Wang J, Zou M, Deng Y, et al The ZAR1 resistosome is a calcium-permeable channel triggering plant immune signaling. Cell. 2021:184(13):3528–3541.e12. 10.1016/j.cell.2021.05.003 33984278

[kiae302-B19] Bohra A , Kilian B, Sivasankar S, Caccamo M, Mba C, McCouch SR, Varshney RK. Reap the crop wild relatives for breeding future crops. Trends Biotechnol. 2022:40(4):412–431. 10.1016/j.tibtech.2021.08.009 34629170

[kiae302-B20] Bokelmann W , Huyskens-Keil S, Ferenczi Z, Stöber S. The role of indigenous vegetables to improve food and nutrition security: experiences from the project HORTINLEA in Kenya (2014–2018). Front Sustain Food Syst. 2022:6:806420. 10.3389/fsufs.2022.806420

[kiae302-B21] Borhan MH , Van De Wouw AP, Larkan NJ. Molecular interactions between *Leptosphaeria maculans* and *Brassica* species. Annu Rev Phytopathol. 2022:60(1):237–257. 10.1146/annurev-phyto-021621-120602 35576591

[kiae302-B22] Campanelli G , Sestili S, Acciarri N, Montemurro F, Palma D, Leteo F, Beretta M. Multi-parental advances generation inter-cross population, to develop organic tomato genotypes by participatory plant breeding. Agronomy. 2019:9(3):119. 10.3390/agronomy9030119

[kiae302-B23] Cao JY , Xu YP, Zhao L, Li SS, Cai XZ. Tight regulation of the interaction between *Brassica napus* and *Sclerotinia sclerotiorum* at the microRNA level. Plant Mol Biol. 2016:92(1-2):39–55. 10.1007/s11103-016-0494-3 27325118

[kiae302-B24] Castillejo MÁ , Fondevilla-Aparicio S, Fuentes-Almagro C, Rubiales D. Quantitative analysis of target peptides related to resistance against *Ascochyta* blight (*Peyronellaea pinodes*) in pea. J Proteome Res. 2020:19(3):1000–1012. 10.1021/acs.jproteome.9b00365 32040328

[kiae302-B25] Chandrasekaran J , Brumin M, Wolf D, Leibman D, Klap C, Pearlsman M, Sherman A, Arazi T, Gal-On A. Development of broad virus resistance in non-transgenic cucumber using CRISPR/Cas9 technology. Mol Plant Pathol. 2016:17(7):1140–1153. 10.1111/mpp.12375 26808139 PMC6638350

[kiae302-B26] Chen YL , Lee CY, Cheng KT, Chang WH, Huang RN, Nam HG, Chen YR. Quantitative peptidomics study reveals that a wound-induced peptide from PR-1 regulates immune signaling in tomato. Plant Cell. 2014:26(10):4135–4148. 10.1105/tpc.114.131185 25361956 PMC4247587

[kiae302-B27] Cheng W , Lin M, Chu M, Xiang G, Guo J, Jiang Y, Guan D, He S. RNAi-based gene silencing of RXLR effectors protects plants against the oomycete pathogen *Phytophthora capsici*. Mol Plant Microbe Interact. 2022:35(6):440–449. 10.1094/MPMI-12-21-0295-R 35196108

[kiae302-B28] Chidambara B , Elangovan D, Avverahally ST, Reddy KM, Kundapura R. Identification of circular RNAs in resistant tomato genotype inresponse to ToLCBaV infection. J Hortic Sci. 2022:17(2):496–504. 10.24154/jhs.v17i2.1520

[kiae302-B29] Christopoulou M , Wo SRC, Kozik A, McHale LK, Truco MJ, Wroblewski T, Michelmore RW. Genome-wide architecture of disease resistance genes in lettuce. G3: Genes, Genomes, Genetics. 2015:5(12):2655–2669. 10.1534/g3.115.020818 26449254 PMC4683639

[kiae302-B30] Corwin JA , Kliebenstein DJ. Quantitative resistance: more than just perception of a pathogen. Plant Cell. 2017:29(4):655–665. 10.1105/tpc.16.00915 28302676 PMC5435431

[kiae302-B31] Cowger C , Brown JKM. Durability of quantitative resistance in crops: greater than we know? Annu Rev Phytopathol. 2019:57(1):253–277. 10.1146/annurev-phyto-082718-100016 31206351

[kiae302-B32] Cui C , Wang JJ, Zhao JH, Fang YY, He XF, Guo HS, Duan CG. A *Brassica* miRNA regulates plant growth and immunity through distinct modes of action. Mol Plant. 2020:13(2):231–245. 10.1016/j.molp.2019.11.010 31794845

[kiae302-B33] Dong OX , Ronald PC. Genetic engineering for disease resistance in plants: recent progress and future perspectives. Plant Physiol. 2019:180(1):26–38. 10.1104/pp.18.01224 30867331 PMC6501101

[kiae302-B34] Edwards D , Batley J. Graph pangenomes find missing heritability. Nat Genet. 2022:54(7):919–920. 10.1038/s41588-022-01099-8 35739387

[kiae302-B35] Eizenga JM , Novak AM, Sibbesen JA, Heumos S, Ghaffaari A, Hickey G, Chang X, Seaman JD, Rounthwaite R, Ebler J, et al Pangenome graphs. Annu Rev Genomics Hum Genet. 2020:21(1):139–162. 10.1146/annurev-genom-120219-080406 32453966 PMC8006571

[kiae302-B36] FAO – Food and Agriculture Organization (2023). FAOSTAT. https://www.fao.org/faostat. Accessed October 28, 2023.

[kiae302-B37] Fei Q , Xia R, Meyers BC. Phased, secondary, small interfering RNAs in posttranscriptional regulatory networks. Plant Cell. 2013:25(7):2400–2415. 10.1105/tpc.113.114652 23881411 PMC3753373

[kiae302-B38] Fernandez CGT , Nestor BJ, Danilevicz MF, Gill M, Petereit J, Bayer PE, Finnegan PM, Batley J, Edwards D. Pangenomes as a resource to accelerate breeding of under-utilised crop species. Int J Mol Sci. 2022:23(5):2671. 10.3390/ijms23052671 35269811 PMC8910360

[kiae302-B39] Gao C . Genome engineering for crop improvement and future agriculture. Cell. 2021:184(6):1621–1635. 10.1016/j.cell.2021.01.005 33581057

[kiae302-B40] Golicz AA , Bayer PE, Barker GC, Edger PP, Kim HR, Martinez PA, Chan CKK, Severn-Ellis A, McCombie WR, Parkin IAP, et al The pangeome of an agronomically important crop plant *Brassica oleracea*. Nat Commun. 2016:7(1):13390. 10.1038/ncomms13390 27834372 PMC5114598

[kiae302-B41] Golicz AA , Bayer PE, Bhalla PL, Batley J, Edwards D. Pangenomics comes of age: from bacteria to plant and animal applications. Trends Genet. 2020:36(2):132–145. 10.1016/j.tig.2019.11.006 31882191

[kiae302-B42] Goncalves-Vidigal MC , Gilio TAS, Valentini G, Vaz-Bisneta M, Vidigal Filho PS, Song Q, Oblessuc PR, Melotto M. New andean source of resistance to anthracnose and angular leaf spot: fine-mapping of disease-resistance genes in California dark red kidney common bean cultivar. PLoS One. 2020:15(6):e0235215. 10.1371/journal.pone.0235215 32598372 PMC7323968

[kiae302-B43] Grech-Baran M , Witek K, Szajko K, Witek AI, Morgiewicz K, Wasilewicz-Flis I, Jakuczun H, Marczewski W, Jones JDG, Hennig J. Extreme resistance to *Potato virus Y* in potato carrying the *Rysto* gene is mediated by a TIR-NLR immune receptor. Plant Biotechnol J. 2020:18(3):655–667. 10.1111/pbi.13230 31397954 PMC7004898

[kiae302-B44] Gupta SK , Thind TS. Disease problems in vegetable production. 2nd ed. Jodhpur, India: Scientific Publishers (India); 2018.

[kiae302-B45] He H , Ge L, Li Z, Zhou X, Li F. Pepino mosaic virus antagonizes plant m6A modification by promoting the autophagic degradation of the m6A writer HAKAI. aBIOTECH. 2023:4(2):83–96. 10.1007/s42994-023-00097-6 37581026 PMC10423194

[kiae302-B46] He Y , Li L, Yao Y, Li Y, Zhang H, Fan M. Transcriptome-wide N6-methyladenosine (m6A) methylation in watermelon under CGMMV infection. BMC Plant Biol. 2021:21(1):516. 10.1186/s12870-021-03289-8 34749644 PMC8574010

[kiae302-B47] Hickey G , Monlong J, Ebler J, Novak AM, Eizenga JM, Gao Y, Human Pangenome Reference Consortium, Marschall T, Li H, Paten B. Pangenome graph construction from genome alignments with Minigraph-Cactus. Nat Biotechnol. 2024:42(4):663–673. 10.1038/s41587-023-01793-w 37165083 PMC10638906

[kiae302-B48] Holdsworth WL , LaPlant KE, Bell DC, Jahn MM, Mazourek M. Cultivar-based introgression mapping reveals wild species-derived *Pm-0*, the major powdery mildew resistance locus in squash. PLoS One. 2016:11(12):e0167715. 10.1371/journal.pone.0167715 27936008 PMC5147965

[kiae302-B49] Hou X , Cui J, Liu W, Jiang N, Zhou X, Qi H, Meng J, Luan Y. LncRNA39026 enhances tomato resistance to *Phytophthora infestans* by decoying miR168a and inducing *PR* gene expression. Phytopathology. 2020:110(4):873–880. 10.1094/PHYTO-12-19-0445-R 31876247

[kiae302-B50] Hu Z , Zhang H, Shi K. Plant peptides in plant defense responses. Plant Signal Behav. 2018:13(8):e1475175. 10.1080/15592324.2018.1475175 30067449 PMC6149413

[kiae302-B51] Huang Z , Qiao F, Yang B, Liu J, Liu Y, Wulff BBH, Hu P, Lv Z, Zhang R, Chen P, et al Genome-wide identification of the NLR gene family in *Haynaldia villosa* by SMRT-RenSeq. BMC Genomics. 2022:23(1):1–23. 10.1186/s12864-022-08334-w 35144544 PMC8832786

[kiae302-B52] Hübner S . Are we there yet? Driving the road to evolutionary graph-pangenomics. Curr Opin Plant Biol. 2022:66:102195. 10.1016/j.pbi.2022.102195 35217472

[kiae302-B53] Javed K , Wang Y, Javed H, Humayun T, Humayun A. Hrip1 induces systemic resistance against bean aphid (*Megoura japonica* Matsumura) in common beans (*Phaseolus vulgaris* L.). Microorganisms. 2022:10(6):1080. 10.3390/microorganisms10061080 35744596 PMC9227054

[kiae302-B54] Jiang N , Cui J, Shi Y, Yang G, Zhou X, Hou X, Meng J, Luan Y. Tomato lncRNA23468 functions as a competing endogenous RNA to modulate *NBS-LRR* genes by decoying miR482b in the tomato-*Phytophthora infestans* interaction. Hortic Res. 2019:6(1):28. 10.1038/s41438-018-0096-0 30729018 PMC6355781

[kiae302-B55] Jones JDG , Dangl JL. The plant immune system. Nature. 2006:444(7117):323–329. 10.1038/nature05286 17108957

[kiae302-B56] Jones DA , Thomas CM, Hammond-Kosack KE, Balint-Kurti PJ, Jones JDG. Isolation of the tomato *Cf-9* gene for resistance to *Cladosporium fulvum* by transposon tagging. Science (1979). 1994:266:789–793. 10.1126/science.7973631 7973631

[kiae302-B57] Jones JDG , Vance RE, Dangl JL. Intracellular innate immune surveillance devices in plants and animals. Science (1979). 2016:354:aaf6395. 10.1126/science.aaf6395 27934708

[kiae302-B58] Joshi RK , Megha S, Basu U, Rahman MH, Kav NNV. Genome wide identification and functional prediction of long non-coding RNAs responsive to *Sclerotinia sclerotiorum* infection in *Brassica napus*. PLoS One. 2016:11(7):e0158784. 10.1371/journal.pone.0158784 27388760 PMC4936718

[kiae302-B59] Jupe F , Witek K, Verweij W, Śliwka J, Pritchard L, Etherington GJ, Maclean D, Cock PJ, Leggett RM, Bryan GJ, et al Resistance gene enrichment sequencing (RenSeq) enables reannotation of the NB-LRR gene family from sequenced plant genomes and rapid mapping of resistance loci in segregating populations. Plant J. 2013:76(3):530–544. 10.1111/tpj.12307 23937694 PMC3935411

[kiae302-B60] Kale SM , Schulthess AW, Padmarasu S, Boeven PHG, Schacht J, Himmelbach A, Steuernagel B, Wulff BBH, Reif JC, Stein N, et al A catalogue of resistance gene homologs and a chromosome-scale reference sequence support resistance gene mapping in winter wheat. Plant Biotechnol J. 2022:20(9):1730–1742. 10.1111/pbi.13843 35562859 PMC9398310

[kiae302-B61] Klessig DF , Choi HW, Dempsey DA. Systemic acquired resistance and salicylic acid: past, present, and future. Mol Plant Microbe Interact. 2018:31(9):871–888. 10.1094/MPMI-03-18-0067-CR 29781762

[kiae302-B62] Kumar K , Mandal SN, Neelam K, de los Reyes BG. MicroRNA-mediated host defense mechanisms against pathogens and herbivores in rice: balancing gains from genetic resistance with trade-offs to productivity potential. BMC Plant Biol. 2022:22(1):351–316. 10.1186/s12870-022-03723-5 35850632 PMC9290239

[kiae302-B63] Lan M , Li G, Hu J, Yang H, Zhang L, Xu X, Liu J, He J, Sun R. iTRAQ-based quantitative analysis reveals proteomic changes in Chinese cabbage (*Brassica rapa* L.) in response to *Plasmodiophora brassicae* infection. Sci Rep. 2019:9(1):12058. 10.1038/s41598-019-48608-0 31427711 PMC6700187

[kiae302-B64] Lee Y-R , Siddique MI, Kim D-S, Lee ES, Han K, Kim S-G, Lee H-E. CRISPR/Cas9-mediated gene editing to confer turnip mosaic virus (TuMV) resistance in Chinese cabbage (*Brassica rapa*). Hortic Res. 2023:10(6):uhad078. 10.1093/hr/uhad078 37323233 PMC10261878

[kiae302-B65] Li H , Wang S, Chai S, Yang Z, Zhang Q, Xin H, Xu Y, Lin S, Chen X, Yao Z, et al Graph-based pan-genome reveals structural and sequence variations related to agronomic traits and domestication in cucumber. Nat Commun. 2022:13(1):682. 10.1038/s41467-022-28362-0 35115520 PMC8813957

[kiae302-B66] Li X , Xu B, Xu J, Li Z, Jiang C, Zhou Y, Yang Z, Deng M, Lv J, Zhao K. Tomato-thaumatin-like protein genes *Solyc08g080660* and *Solyc08g080670* confer resistance to five soil-borne diseases by enhancing β-1,3-glucanase activity. Genes (Basel). 2023:14(8):1622. 10.3390/genes14081622 37628673 PMC10454901

[kiae302-B67] Liang L , Zhang J, Xiao J, Li X, Xie Y, Tan H, Song X, Zhu L, Xue X, Xu L, et al Genome and pan-genome assembly of asparagus bean (*Vigna unguiculata* ssp. *sesquipedialis*) reveal the genetic basis of cold adaptation. Front Plant Sci. 2022:13:1059804. 10.3389/fpls.2022.1059804 36589110 PMC9802904

[kiae302-B68] Lin X , Armstrong M, Baker K, Wouters D, Visser RGF, Wolters PJ, Hein I, Vleeshouwers VGAA. RLP/k enrichment sequencing; a novel method to identify receptor-like protein (RLP) and receptor-like kinase (RLK) genes. New Phytol. 2020:227(4):1264–1276. 10.1111/nph.16608 32285454 PMC7383770

[kiae302-B69] Lin X , Jia Y, Heal R, Prokchorchik M, Sindalovskaya M, Olave-Achury A, Makechemu M, Fairhead S, Noureen A, Heo J, et al *Solanum americanum* genome-assisted discovery of immune receptors that detect potato late blight pathogen effectors. Nat Genet. 2023:55(9):1579–1588. 10.1038/s41588-023-01486-9 37640880 PMC10484786

[kiae302-B70] Lin X , Olave-Achury A, Heal R, Pais M, Witek K, Ahn HK, Zhao H, Bhanvadia S, Karki HS, Song T, et al A potato late blight resistance gene protects against multiple *Phytophthora* species by recognizing a broadly conserved RXLR-WY effector. Mol Plant. 2022:15(9):1457–1469. 10.1016/j.molp.2022.07.012 35915586

[kiae302-B71] Lin X , Zhang Y, Kuang H, Chen J. Frequent loss of lineages and deficient duplications accounted for low copy number of disease resistance genes in Cucurbitaceae. BMC Genomics. 2013:14(1):335. 10.1186/1471-2164-14-335 23682795 PMC3679737

[kiae302-B72] Liu H , Nwafor CC, Piao Y, Li X, Zhan Z, Piao Z. Identification and characterization of circular RNAs in *Brassica rapa* in response to *Plasmodiophora brassicae*. Int J Mol Sci. 2022:23(10):5369. 10.3390/ijms23105369 35628175 PMC9141718

[kiae302-B73] Liu K , Xu H, Gao X, Lu Y, Wang L, Ren Z, Chen C. Pan-genome analysis of *TIFY* gene family and functional analysis of *CsTIFY* genes in cucumber. Int J Mol Sci. 2024:25(1):185. 10.3390/ijms25010185 PMC10778933 38203357

[kiae302-B74] Mackenzie A , Norman M, Gessese M, Chen C, Sørensen C, Hovmøller M, Ma L, Forrest K, Hickey L, Bariana H, et al Wheat stripe rust resistance locus *YR63* is a hot spot for evolution of defence genes—a pangenome discovery. BMC Plant Biol. 2023:23(1):590. 10.1186/s12870-023-04576-2 38008766 PMC10680240

[kiae302-B75] Mangino G , Arrones A, Plazas M, Pook T, Prohens J, Gramazio P, Vilanova S. Newly developed MAGIC population allows identification of strong associations and candidate genes for anthocyanin pigmentation in eggplant. Front Plant Sci. 2022:13:847789. 10.3389/fpls.2022.847789 35330873 PMC8940277

[kiae302-B76] Martin GB , Brommonschenkel SH, Chunwongse J, Frary A, Ganal MW, Spivey R, Wu T, Earle ED, Tanksley SD. Map-based cloning of a protein kinase gene conferring disease resistance in tomato. Science (1979). 1993:262:1432–1436. 10.1126/science.7902614 7902614

[kiae302-B77] Meline V , Hendrich CG, Truchon AN, Caldwell D, Hiles R, Leuschen-Kohl R, Tran T, Mitra RM, Allen C, Iyer-Pascuzzi AS. Tomato deploys defence and growth simultaneously to resist bacterial wilt disease. Plant Cell Environ. 2023:46(10):3040–3058. 10.1111/pce.14456 36213953

[kiae302-B78] Miao Z , Zhang T, Xie B, Qi Y, Ma C. Evolutionary implications of the RNA N6-methyladenosine methylome in plants. Mol Biol Evol. 2022:39(1):msab299. 10.1093/molbev/msab299 34633447 PMC8763109

[kiae302-B79] Mitter N , Worrall EA, Robinson KE, Li P, Jain RG, Taochy C, Fletcher SJ, Carroll BJ, Lu GQ, Xu ZP. Clay nanosheets for topical delivery of RNAi for sustained protection against plant viruses. Nat Plants. 2017:3(2):16207–16210. 10.1038/nplants.2016.207 28067898

[kiae302-B80] Monino-Lopez D , Nijenhuis M, Kodde L, Kamoun S, Salehian H, Schentsnyi K, Stam R, Lokossou A, Abd-El-Haliem A, Visser RGF, et al Allelic variants of the NLR protein Rpi-chc1 differentially recognize members of the *Phytophthora infestans* PexRD12/31 effector superfamily through the leucine-rich repeat domain. Plant J. 2021:107(1):182–197. 10.1111/tpj.15284 33882622 PMC8362081

[kiae302-B81] Muffato M , Louis A, Nguyen NTT, Lucas J, Berthelot C, Crollius HR. Reconstruction of hundreds of reference ancestral genomes across the eukaryotic kingdom. Nat Ecol Evol. 2023:7(3):355–366. 10.1038/s41559-022-01956-z 36646945 PMC9998269

[kiae302-B82] Naguib DM , Alzandi AA, Shamkh IM, Reyad NEHA. Fabatin induce defense-related enzymes in cucumber against soil born pathogen, *Fusarium oxysporum*. Rhizosphere. 2021:19:100381. 10.1016/j.rhisph.2021.100381

[kiae302-B83] Ngou BPM , Ahn HK, Ding P, Jones JDG. Mutual potentiation of plant immunity by cell-surface and intracellular receptors. Nature. 2021:592(7852):110–115. 10.1038/s41586-021-03315-7 33692545

[kiae302-B84] Niks RE , Qi X, Marcel TC. Quantitative resistance to biotrophic filamentous plant pathogens: concepts, misconceptions, and mechanisms. Annu Rev Phytopathol. 2015:53(1):445–470. 10.1146/annurev-phyto-080614-115928 26047563

[kiae302-B85] Niu Y , Liu Q, He Z, Raman R, Wang H, Long X, Qin H, Raman H, Parkin IAP, Bancroft I, et al A *Brassica carinata* pan-genome platform for *Brassica* crop improvement. Plant Commun. 2024:5(1):100725. 10.1016/j.xplc.2023.100725 37803826 PMC10811369

[kiae302-B86] Oh S , Kim S, Park HJ, Kim MS, Seo MK, Wu CH, Lee HA, Kim HS, Kamoun S, Choi D. Nucleotide-binding leucine-rich repeat network underlies nonhost resistance of pepper against the Irish potato famine pathogen *Phytophthora infestans*. Plant Biotechnol J. 2023:21(7):1361–1372. 10.1111/pbi.14039 36912620 PMC10281606

[kiae302-B87] Ou L , Li D, Lv J, Chen W, Zhang Z, Li X, Yang B, Zhou S, Yang S, Li W, et al Pan-genome of cultivated pepper (*Capsicum*) and its use in gene presence–absence variation analyses. New Phytol. 2018:220(2):360–363. 10.1111/nph.15413 30129229

[kiae302-B88] Ouyang S-Q , Ji H-M, Feng T, Luo S-J, Cheng L, Wang N. Artificial trans-kingdom RNAi of *FolRDR1* is a potential strategy to control tomato wilt disease. PLoS Pathog. 2023:19(6):e1011463. 10.1371/journal.ppat.1011463 37339156 PMC10313012

[kiae302-B89] Paluchowska P , Śliwka J, Yin Z. Late blight resistance genes in potato breeding. Planta. 2022:255(6):127. 10.1007/s00425-022-03910-6 35576021 PMC9110483

[kiae302-B90] Pant P , Kaur J. Spray-induced gene silencing of *SsOah1* and *SsCyp51* confers protection to *Nicotiana benthamiana* and *Brassica juncea* against *Sclerotinia sclerotiorum*. Physiol Mol Plant Pathol. 2023:127:102109. 10.1016/j.pmpp.2023.102109

[kiae302-B91] Parajuli R , Thoma G, Matlock MD. Environmental sustainability of fruit and vegetable production supply chains in the face of climate change: a review. Sci Total Environ. 2019:650:2863–2879. 10.1016/j.scitotenv.2018.10.019 30373063

[kiae302-B92] Parra L , Nortman K, Sah A, Truco MJ, Ochoa O, Michelmore R. Identification and mapping of new genes for resistance to downy mildew in lettuce. Theor Appl Genet. 2021a:134(2):519–528. 10.1007/s00122-020-03711-z 33128618 PMC7843477

[kiae302-B93] Parra L , Simko I, Michelmore RW. Identification of major quantitative trait loci controlling field resistance to downy mildew in cultivated lettuce (*Lactuca sativa*). Phytopathology. 2021b:111(3):541–547. 10.1094/PHYTO-08-20-0367-R 33141649

[kiae302-B94] Pascual L , Desplat N, Huang BE, Desgroux A, Bruguier L, Bouchet JP, Le QH, Chauchard B, Verschave P, Causse M. Potential of a tomato MAGIC population to decipher the genetic control of quantitative traits and detect causal variants in the resequencing era. Plant Biotechnol J. 2015:13(4):565–577. 10.1111/pbi.12282 25382275

[kiae302-B95] Pink H , Talbot A, Graceson A, Graham J, Higgins G, Taylor A, Jackson AC, Truco M, Michelmore R, Yao C, et al Identification of genetic loci in lettuce mediating quantitative resistance to fungal pathogens. Theor Appl Genet. 2022:135(7):2481–2500. 10.1007/s00122-022-04129-5 35674778 PMC9271113

[kiae302-B96] Pokhrel B . Effects of environmental factors on crop diseases development. J Plant Pathol Microbiol. 2021:12:553. 10.35248/2157-7471.21.12.553

[kiae302-B97] Poland JA , Balint-Kurti PJ, Wisser RJ, Pratt RC, Nelson RJ. Shades of gray: the world of quantitative disease resistance. Trends Plant Sci. 2009:14(1):21–29. 10.1016/j.tplants.2008.10.006 19062327

[kiae302-B98] Pruitt RN , Locci F, Wanke F, Zhang L, Saile SC, Joe A, Karelina D, Hua C, Fröhlich K, Wan WL, et al The EDS1–PAD4–ADR1 node mediates *Arabidopsis* pattern-triggered immunity. Nature. 2021:598(7881):495–499. 10.1038/s41586-021-03829-0 34497423

[kiae302-B99] Qiao L , Lan C, Capriotti L, Ah-Fong A, Nino Sanchez J, Hamby R, Heller J, Zhao H, Glass NL, Judelson HS, et al Spray-induced gene silencing for disease control is dependent on the efficiency of pathogen RNA uptake. Plant Biotechnol J. 2021:19(9):1756–1768. 10.1111/pbi.13589 33774895 PMC8428832

[kiae302-B100] Qu Q , Liu N, Su Q, Liu X, Jia H, Liu Y, Sun M, Cao Z, Dong J. MicroRNAs involved in the trans-kingdom gene regulation in the interaction of maize kernels and *Fusarium verticillioides*. Int J Biol Macromol. 2023:242:125046. 10.1016/j.ijbiomac.2023.125046 37245767

[kiae302-B101] Rahman MM , Porter LD, Ma Y, Coyne CJ, Zheng P, Chaves-Cordoba B, Naidu RA. Resistance in pea (*Pisum sativum*) genetic resources to the pea aphid, *Acyrthosiphon pisum*. Entomol Exp Appl. 2023:171(6):435–448. 10.1111/eea.13296

[kiae302-B102] Rajpal VR , Singh A, Kathpalia R, Kr Thakur R, Khan MK, Pandey A, Hamurcu M, Raina SN. The prospects of gene introgression from crop wild relatives into cultivated lentil for climate change mitigation. Front Plant Sci. 2023:14:1127239. 10.3389/fpls.2023.1127239 36998696 PMC10044020

[kiae302-B103] Razzaq A , Kaur P, Akhter N, Wani SH, Saleem F. Next-generation breeding strategies for climate-ready crops. Front Plant Sci. 2021:12:620420. 10.3389/fpls.2021.620420 34367194 PMC8336580

[kiae302-B104] Richard B , Qi A, Fitt BDL. Control of crop diseases through integrated crop management to deliver climate-smart farming systems for low- and high-input crop production. Plant Pathol. 2022:71(1):187–206. 10.1111/ppa.13493

[kiae302-B105] Rojas DKJ , Sedano JCS, Ballvora A, Léon J, Vásquez TM. Novel organ-specific genetic factors for quantitative resistance to late blight in potato. PLoS One. 2019:14(7):e0213818. 10.1371/journal.pone.0213818 31310605 PMC6634379

[kiae302-B106] Savary S , Willocquet L, Pethybridge SJ, Esker P, McRoberts N, Nelson A. The global burden of pathogens and pests on major food crops. Nat Ecol Evol. 2019:3(3):430–439. 10.1038/s41559-018-0793-y 30718852

[kiae302-B107] Schwartz SH , Hendrix B, Hoffer P, Sanders RA, Zheng W. Carbon dots for efficient small interfering RNA delivery and gene silencing in plants. Plant Physiol. 2020:184(2):647–657. 10.1104/pp.20.00733 32764133 PMC7536711

[kiae302-B108] Shaw RK , Shen Y, Yu H, Sheng X, Wang J, Gu H. Multi-omics approaches to improve clubroot resistance in Brassica with a special focus on *Brassica oleracea* L. Int J Mol Sci. 2022:23(16):9280. 10.3390/ijms23169280 36012543 PMC9409056

[kiae302-B109] Shen X , Yan Z, Wang X, Wang Y, Arens M, Du Y, Visser RGF, Kormelink R, Bai Y, Wolters AMA. The NLR protein encoded by the resistance gene *Ty-2* is triggered by the replication-associated protein Rep/C1 of tomato yellow leaf curl virus. Front Plant Sci. 2020:11:545306. 10.3389/fpls.2020.545306 33013967 PMC7511541

[kiae302-B110] Shrestha S , Michael VN, Fu Y, Meru G. Genetic loci associated with resistance to zucchini yellow mosaic virus in squash. Plants. 2021:10(9):1935. 10.3390/plants10091935 34579467 PMC8465829

[kiae302-B111] Simko I , Puri KD, Dhar N, Peng H, Subbarao KV. Mapping quantitative trait loci for lettuce resistance to *Verticillium dahliae* Race 3, plant development, and leaf color using an ultra-high-density bin map constructed from F2 progeny. PhytoFrontiers. 2022:2(3):257–267. 10.1094/PHYTOFR-11-21-0078-R

[kiae302-B112] Slavin JL , Lloyd B. Health benefits of fruits and vegetables. Adv Nutr. 2012:3(4):506–516. 10.3945/an.112.002154 22797986 PMC3649719

[kiae302-B113] Song L , Fang Y, Chen L, Wang J, Chen X. Role of non-coding RNAs in plant immunity. Plant Commun. 2021:2(3):100180. 10.1016/j.xplc.2021.100180 34027394 PMC8132121

[kiae302-B114] Song X-S , Gu K-X, Duan X-X, Xiao X-M, Hou Y-P, Duan Y-B, Wang J-X, Yu N, Zhou M-G. Secondary amplification of siRNA machinery limits the application of spray-induced gene silencing. Mol Plant Pathol. 2018:19(12):2543–2560. 10.1111/mpp.12728 30027625 PMC6638038

[kiae302-B115] Stare T , Ramšak Ž, Križnik M, Gruden K. Multiomics analysis of tolerant interaction of potato with potato virus Y. Sci Data. 2019:6(1):250. 10.1038/s41597-019-0216-1 31673114 PMC6823367

[kiae302-B116] St. Clair DA . Quantitative disease resistance and quantitative resistance loci in breeding. Annu Rev Phytopathol. 2010:48(1):247–268. 10.1146/annurev-phyto-080508-081904 19400646

[kiae302-B117] Steuernagel B , Periyannan SK, Hernández-Pinzón I, Witek K, Rouse MN, Yu G, Hatta A, Ayliffe M, Bariana H, Jones JDG, et al Rapid cloning of disease-resistance genes in plants using mutagenesis and sequence capture. Nat Biotechnol. 2016:34(6):652–655. 10.1038/nbt.3543 27111722

[kiae302-B118] Steuernagel B , Witek K, Krattinger SG, Ramirez-Gonzalez RH, Schoonbeek H-J, Yu G, Baggs E, Witek AI, Yadav I, Krasileva KV, et al The NLR-annotator tool enables annotation of the intracellular immune receptor repertoire. Plant Physiol. 2020:183(2):468–482. 10.1104/pp.19.01273 32184345 PMC7271791

[kiae302-B119] Sthapit Kandel J , Sandoya GV, Zhou W, Read QD, Mou B, Simko I. Identification of quantitative trait loci associated with bacterial leaf spot resistance in baby leaf lettuce. Plant Dis. 2022:106(10):2583–2590. 10.1094/PDIS-09-21-2087-RE 35285269

[kiae302-B120] Summanwar A , Basu U, Kav NNV, Rahman H. Identification of lncRNAs in response to infection by *Plasmodiophora brassicae* in *Brassica napus* and development of lncRNA-based SSR markers. Genome. 2021:64(5):547–566. 10.1139/gen-2020-0062 33170735

[kiae302-B121] Sun S , Deng D, Wang Z, Duan C, Wu X, Wang X, Zong X, Zhu Z. A novel *er1* allele and the development and validation of its functional marker for breeding pea (*Pisum sativum* L.) resistance to powdery mildew. Theor Appl Genet. 2016:129(5):909–919. 10.1007/s00122-016-2671-9 26801335

[kiae302-B122] Tang D , Jia Y, Zhang J, Li H, Cheng L, Wang P, Bao Z, Liu Z, Feng S, Zhu X, et al Genome evolution and diversity of wild and cultivated potatoes. Nature. 2022:606(7914):535–541. 10.1038/s41586-022-04822-x 35676481 PMC9200641

[kiae302-B123] Tetorya M , Li H, Djami-Tchatchou AT, Buchko GW, Czymmek KJ, Shah DM. Plant defensin MtDef4-derived antifungal peptide with multiple modes of action and potential as a bio-inspired fungicide. Mol Plant Pathol. 2023:24(8):896–913. 10.1111/mpp.13336 37036170 PMC10346373

[kiae302-B124] Thomma Bphj , Nürnberger T, Joosten Mhaj. Of PAMPs and effectors: The blurred PTI-ETI dichotomy. Plant Cell. 2011:23(1):4–15. 10.1105/tpc.110.082602 21278123 PMC3051239

[kiae302-B125] Tian H , Wu Z, Chen S, Ao K, Huang W, Yaghmaiean H, Sun T, Xu F, Zhang Y, Wang S, et al Activation of TIR signalling boosts pattern-triggered immunity. Nature. 2021:598(7881):500–503. 10.1038/s41586-021-03987-1 34544113

[kiae302-B126] Tirnaz S , Zandberg J, Thomas WJW, Marsh J, Edwards D, Batley J. Application of crop wild relatives in modern breeding: an overview of resources, experimental and computational methodologies. Front Plant Sci. 2022:13:1008904. 10.3389/fpls.2022.1008904 36466237 PMC9712971

[kiae302-B127] Toda N , Rustenholz C, Baud A, Le Paslier MC, Amselem J, Merdinoglu D, Faivre-Rampant P. NLGenomeSweeper: a tool for genome-wide NBS-LRR resistance gene identification. Genes (Basel). 2020:11(3):333. 10.3390/genes11030333 32245073 PMC7141099

[kiae302-B128] Tonini P , Odina PM, Orsini F, Durany XG. Economic benefit and social impact derived by a food loss prevention strategy in the vegetable sector: a spatial and temporal analysis at the regional level. Front Sustain Food Syst. 2022:6:1043591. 10.3389/fsufs.2022.1043591

[kiae302-B129] Usadel B . *Solanaceae* pangenomes are coming of graphical age to bring heritability back. aBIOTECH. 2022:3(4):233–236. 10.1007/s42994-022-00087-0 36533266 PMC9755766

[kiae302-B130] Van de Weyer AL , Monteiro F, Furzer OJ, Nishimura MT, Cevik V, Witek K, Jones JDG, Dangl JL, Weigel D, Bemm F. A species-wide inventory of NLR genes and alleles in *Arabidopsis thaliana*. Cell. 2019:178(5):1260–1272.e14. 10.1016/j.cell.2019.07.038 31442410 PMC6709784

[kiae302-B131] Wang S , Liu L, Mi X, Zhao S, An Y, Xia X, Guo R, Wei C. Multi-omics analysis to visualize the dynamic roles of defense genes in the response of tea plants to gray blight. Plant J. 2021a:106(3):862–875. 10.1111/tpj.15203 33595875

[kiae302-B132] Wang Y , Qi C, Luo Y, Zhang F, Dai Z, Li M, Qu S. Identification and mapping of *CpPM10.1*, a major gene involved in powdery mildew (race 2 France of *Podosphaera xanthii*) resistance in zucchini (*Cucurbita pepo* L.). Theor Appl Genet. 2021b:134(8):2531–2545. 10.1007/s00122-021-03840-z 33914112

[kiae302-B133] Wang Y , Tan J, Wu Z, VandenLangenberg K, Wehner TC, Wen C, Zheng X, Owens K, Thornton A, Bang HH, et al STAYGREEN, STAY HEALTHY: a loss-of-susceptibility mutation in the *STAYGREEN* gene provides durable, broad-spectrum disease resistances for over 50 years of US cucumber production. New Phytol. 2019:221(1):415–430. 10.1111/nph.15353 30022503

[kiae302-B134] Wei X , Zhang Y, Zhao Y, Xie Z, Hossain MR, Yang S, Shi G, Lv Y, Wang Z, Tian B, et al Root transcriptome and metabolome profiling reveal key phytohormone-related genes and pathways involved clubroot resistance in *Brassica rapa* L. Front Plant Sci. 2021:12:759623. 10.3389/fpls.2021.759623 34975941 PMC8715091

[kiae302-B135] Weiberg A , Wang M, Lin FM, Zhao H, Zhang Z, Kaloshian I, Huang H-D, Jin H. Fungal small RNAs suppress plant immunity by hijacking host RNA interference pathways. Science (1979). 2013:342:118–123. 10.1126/science.1239705 PMC4096153 24092744

[kiae302-B136] Williamson-Benavides BA , Sharpe RM, Nelson G, Bodah ET, Porter LD, Dhingra A. Identification of root rot resistance QTLs in pea using *Fusarium solani* f. sp. *pisi*-responsive differentially expressed genes. Front Genet. 2021:12:629267. 10.3389/fgene.2021.629267 34421980 PMC8375389

[kiae302-B137] Witek K , Jupe F, Witek AI, Baker D, Clark MD, Jones JDG. Accelerated cloning of a potato late blight-resistance gene using RenSeq and SMRT sequencing. Nat Biotechnol. 2016:34(6):656–660. 10.1038/nbt.3540 27111721

[kiae302-B138] Witek K , Lin X, Karki HS, Jupe F, Witek AI, Steuernagel B, Stam R, van Oosterhout C, Fairhead S, Heal R, et al A complex resistance locus in *Solanum americanum* recognizes a conserved *Phytophthora* effector. Nat Plants. 2021:7(2):198–208. 10.1038/s41477-021-00854-9 33574576 PMC7116783

[kiae302-B139] Wu X , Wang B, Xin Y, Wang Y, Tian S, Wang J, Wu X, Lu Z, Qi X, Xu L, et al Unravelling the genetic architecture of rust resistance in the common bean (*Phaseolus vulgaris* L.) by combining QTL-Seq and GWAS analysis. Plants. 2022:11(7):953. 10.3390/plants11070953 35406934 PMC9002482

[kiae302-B140] Xu J , Xian Q, Wang K, Dong J, Zhang C, Du S, Xu X, Chen X. Screening and identification of candidate *Fusarium* wilt-resistance genes from pumpkin. Hortic Plant J. 2022:8(5):583–592. 10.1016/j.hpj.2021.11.011

[kiae302-B142] Yang H , Luo P. Changes in photosynthesis could provide important insight into the interaction between wheat and fungal pathogens. Int J Mol Sci. 2021:22(16):8865. 10.3390/ijms22168865 34445571 PMC8396289

[kiae302-B141] Yang T , Liu R, Luo Y, Hu S, Wang D, Wang C, Pandey MK, Ge S, Xu Q, Li N, et al Improved pea reference genome and pan-genome highlight genomic features and evolutionary characteristics. Nature Genetics. 2022:54(10):1553–1563. 10.1038/s41588-022-01172-2 36138232 PMC9534762

[kiae302-B143] Yates S , Mikaberidze A, Krattinger SG, Abrouk M, Hund A, Yu K, Studer B, Fouche S, Meile L, Pereira D, et al Precision phenotyping reveals novel loci for quantitative resistance to septoria tritici blotch. Plant Phenomics. 2019:2019:3285904. 10.34133/2019/3285904 33313526 PMC7706307

[kiae302-B144] Yoon Y-J , Venkatesh J, Lee J-H, Kim J, Lee H-E, Kim D-S, Kang B-C. Genome editing of *eIF4E1* in tomato confers resistance to pepper mottle virus. Front Plant Sci. 2020:11:1098. 10.3389/fpls.2020.01098 32849681 PMC7396686

[kiae302-B145] Yuan Y , Bayer PE, Batley J, Edwards D. Current status of structural variation studies in plants. Plant Biotechnol J. 2021b:19(11):2153–2163. 10.1111/pbi.13646 34101329 PMC8541774

[kiae302-B146] Yuan M , Jiang Z, Bi G, Nomura K, Liu M, Wang Y, Cai B, Zhou JM, He SY, Xin XF. Pattern-recognition receptors are required for NLR-mediated plant immunity. Nature. 2021a:592(7852):105–109. 10.1038/s41586-021-03316-6 33692546 PMC8016741

[kiae302-B147] Yue J , Wei Y, Zhao M. The reversible methylation of m6A is involved in plant virus infection. Biology (Basel). 2022:11(2):271. 10.3390/biology11020271 35205137 PMC8869485

[kiae302-B148] Yuen J . Pathogens which threaten food security: *phytophthora infestans*, the potato late blight pathogen. Food Secur. 2021:13(2):247–253. 10.1007/s12571-021-01141-3

[kiae302-B149] Zanini SF , Bayer PE, Wells R, Snowdon RJ, Batley J, Varshney RK, Nguyen HT, Edwards D, Golicz AA. Pangenomics in crop improvement—from coding structural variations to finding regulatory variants with pangenome graphs. Plant Genome. 2022:15(1):e20177. 10.1002/tpg2.20177 34904403 PMC12806906

[kiae302-B150] Zeng J , Gupta VK, Jiang Y, Yang B, Gong L, Zhu H. Cross-kingdom small RNAs among animals, plants and microbes. Cells. 2019:8(4):371. 10.3390/cells8040371 31018602 PMC6523504

[kiae302-B151] Zhang H . Plant latent defense response against compatibility. ISME J. 2023:17(6):787–791. 10.1038/s41396-023-01399-9 36991179 PMC10203107

[kiae302-B152] Zhang B , Su T, Li P, Xin X, Cao Y, Wang W, Zhao X, Zhang D, Yu Y, Li D, et al Identification of long noncoding RNAs involved in resistance to downy mildew in Chinese cabbage. Hortic Res. 2021b:8(1):44. 10.1038/s41438-021-00479-1 33642586 PMC7917106

[kiae302-B153] Zhang Y , Thomas W, Bayer PE, Edwards D, Batley J. Frontiers in dissecting and managing *Brassica* diseases: from reference-based RGA candidate identification to building pan-RGAomes. Int J Mol Sci. 2020:21(23):8964. 10.3390/ijms21238964 33255840 PMC7728316

[kiae302-B154] Zhang K , Zhuang X, Dong Z, Xu K, Chen X, Liu F, He Z. The dynamics of N6-methyladenine RNA modification in interactions between rice and plant viruses. Genome Biol. 2021a:22(1):189. 10.1186/s13059-021-02410-2 34167554 PMC8229379

[kiae302-B155] Zhou F , Emonet A, Dénervaud Tendon V, Marhavy P, Wu D, Lahaye T, Geldner N. Co-incidence of damage and microbial patterns controls localized immune responses in roots. Cell. 2020:180(3):440–453.e18. 10.1016/j.cell.2020.01.013 32032516 PMC7042715

[kiae302-B156] Zhou Y , Zhang Z, Bao Z, Li H, Lyu Y, Zan Y, Wu Y, Cheng L, Fang Y, Wu K, et al Graph pangenome captures missing heritability and empowers tomato breeding. Nature. 2022:606(7914):527–534. 10.1038/s41586-022-04808-9 35676474 PMC9200638

[kiae302-B157] Zhu M , Jiang L, Bai B, Zhao W, Chen X, Li J, Liu Y, Chen Z, Wang B, Wang C, et al The intracellular immune receptor Sw-5b confers broad-ppectrum resistance to tospoviruses through recognition of a conserved 21-amino acid viral effector epitope. Plant Cell. 2017:29(9):2214–2232. 10.1105/tpc.17.00180 28814646 PMC5635987

