# Supplementary material for: Recent advances in the improvement of genetic resistance against disease in vegetable crops
Source: Plant Physiol. 2024 May 26;196(1):32–46. doi: 10.1093/plphys/kiae302 (PMC11376385; doi:10.1093/plphys/kiae302)
Supplement: kiae302_Supplementary_Data [file kiae302_supplementary_data.docx]

**Supplementary Table S1. Overview of the common pathogens that cause disease to vegetable crops.**

| **Microorganism** | **Pathogen** | **Disease** | **Host** |
| --- | --- | --- | --- |
| Bacteria | *Ralstonia solanacearum* | Bacterial wilt | Cucumber, tomato, pepper |
|  | *Xanthomonas* spp. | Bacterial spot | Tomato, pepper |
|  | *Alternaria solani* | Early blight | Tomato, potato |
|  | *Xanthomonas campestris pv. campestris* | Black rot | Brassicas |
|  | *Clavibacter michiganensis pv. michiganensis* | Bacterial canker | Tomato, capsicum, chilli |
|  | *Pseudomonas* spp.*, Erwinia* spp. | Bacterial soft rot | Lettuce, brassicas, cucurbits, tomato, capsicum, potato, sweet potato |
|  | *Seudomonas syringae -* various strains | Bacterial leaf spot/bacterial spot/bacterial blight | Beet, spring onions, leeks, rocket, coriander |
|  | *Pseudomonas syringae pv. syringae* | Bacterial brown spot | Beans |
| Basidiomycete | *Albugo candida* | White blister/white rust | Brassicas |
| Fungus | *Peronospora* spp. | Downy mildew | Cucumber, squash, melon |
|  | *Erysiphales* spp. | Powdery mildew | Cucumber, squash, pumpkin |
|  | *Leptosphaeria maculans* | Blackleg | Brassicas |
|  | *Fusarium oxysporum* | Fusarium wilt | Tomato, pepper, eggplant |
|  | *Verticillium* spp. | Verticillium wilt | Tomato, potato, pepper, eggplant |
|  | *Colletotrichum* spp. | Anthracnose | Tomato, pepper, cucumber, squash |
|  | *Plasmodiophora brassicae* | Clubroot | Brassicas |
|  | *Sclerotinia sclerotiorum*and*S. minor* | Sclerotinia rots | Most vegetable crops |
|  | *Sclerotium rolfsii*and*S. cepivorum* | Sclerotium rots | Beans, beets, carrot, potato, tomato, capsicum |
|  | *Rhizoctonia solani* | Rhizoctonia rots | Lettuce, potato, brassicas, beans, peas, beets, carrots, capsicum |
|  | *Puccinia sorghi, Uromyces appendiculatus* | Rusts | Sweet corn, beans |
|  | *Botrytis cinerea* | Gray mould | Tomato, pepper, cucumber, squash |
| Oomycete | *Phytophthora infestans* | Late blight | Tomato, potato |
|  | *Pythium spp.* | Pythium | Cucurbits, brassicas, lettuce |
| Virus | *Cucumovirus* | Cucumber mosaic virus (CMV) | Cucumber, tomato, pepper |
|  | *Tobamovirus* | Tomato mosaic virus (ToMV) | Tomato, pepper |
|  | *Orthotospovirus* | Tomato spotted wilt virus (TSWV) | Tomato, pepper, eggplant |
|  |  | | |
